# Synthesis of heterocycles based on azomethine ylides from α-amino acids (or amines) and carbonyl compounds

**DOI:** 10.3762/bjoc.22.55

**Published:** 2026-05-13

**Authors:** Ekaterina V Berezhnaya, Alexander I Ponyaev, Vitali M Boitsov, Alexander V Stepakov

**Affiliations:** 1 Saint Petersburg State Institute of Technology (Technical University), Moskovsky prospect 26, 190013, St. Petersburg, Russian Federation; 2 Alferov University, ul. Khlopina 8/3, 194021, St. Petersburg, Russian Federation; 3 Saint Petersburg State University, Universitetskaya nab. 7/9, 199034, St. Petersburg, Russian Federation

**Keywords:** alkenes, α-amino acids, azomethine ylides, carbonyl compounds, catalysis, (3 + 2) cycloaddition, decarboxylation, dipolarophiles, iminoesters, polycyclic compounds, spirocyclic compounds, stereoselectivity

## Abstract

This review focuses on new directions in (3 + 2) cycloaddition of azomethine ylides to alkenes, resulting in the formation of fused or spiro-fused pyrrolidine derivatives with multiple chiral centers under high regio- and stereocontrol. Currently, strategies using azomethine ylides based on imino esters or α-amino acids with a variety of cyclic and acyclic carbonyl compounds dominate. Enantioselective (3 + 2) cycloaddition reactions of azomethine ylides obtained from imino esters, catalyzed by chiral Cu(I,II) and Ag(I) complexes, are widely used. Reactions using α-amino acids proceed, in most cases, without the use of catalysts, with high yields and high stereoselectivity. Electrophilic alkenes of various structures, (hetero)aromatic olefins and benzofulvenes, cyclic and acyclic unsaturated substrates, and fullerenes are useful dipolarophiles. This reaction method allows for the single-step creation of a wide variety of complex polyheterocyclic systems that may be useful for practical applications.

## Introduction

The 1,3-dipolar cycloaddition is one of the most popular pericyclic reactions in organic synthesis, in which a dipole molecule interacts with a dipolarophile, such as an alkene or alkyne, to form a five-membered heterocycle in one step. Currently, this type of reaction allows for the efficient preparation of bi- and polycyclic fused or spiro-linked structures with multiple chiral centers and high regio- and stereocontrol [1–4]. The use of azomethine ylides as dipoles is necessary for the synthesis of pyrrolidine systems, which are often found in natural products and are important structural fragments of pharmaceuticals [5]. This method is also used to obtain pyrrolizidine derivatives, which are the structural basis of pyrrolizidine alkaloids with diverse biological activity [6–8].

**Scheme 1:**
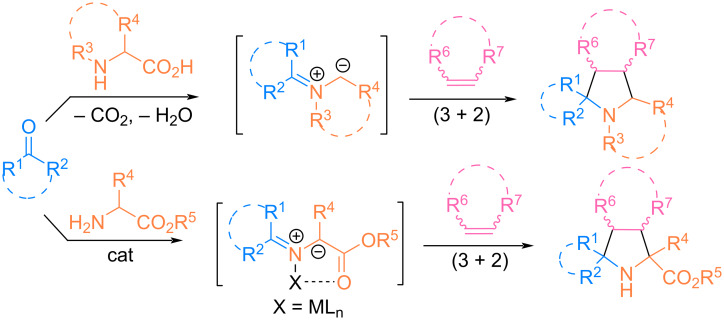
Strategies for the preparation of pyrrolidine derivatives by (3 + 2) cycloaddition of azomethine ylides to alkenes.

An analysis of experimental and review articles showed that (3 + 2) cycloaddition reactions are carried out, in most cases, using two main strategies for obtaining azomethine ylides, depending on the amino acid derivative used: a free amino acid or its ester (Scheme 1).

In both cases, condensation of amino acids with carbonyl compounds occurs; however, in the first case, decarboxylation occurs with the in situ formation of azomethine ylides, which subsequently undergo cycloaddition to unsaturated substrates. The advantage of the first strategy is that the synthesis is often carried out under mild conditions without the use of a catalyst, and despite this, the resulting cycloadducts exhibit pronounced regio- and stereoselectivity [9–11].

In the second case, the generation of azomethine ylides from iminoesters is often realized using chiral metal complexes of Ag(I), Cu(I/II), Zn(II), Ni(II) with ligands of the Segphos, Fesulfos, or Biphamphos type [12–15]. Such catalytic systems control the enantioselectivity of cycloaddition and allow the preparation of enantioenriched pyrrolidines containing several stereocenters in high yields.

Previously published reviews [16–19] focused on decarboxylative reactions of 1,3-dipolar cycloaddition of azomethine ylides from isatin, acenaphthenequinone or ninhydrin and amino acids to unsaturated 2π-electron substrates. In review works [20–21] the synthesis of spirooxindole-pyrrolizidine derivatives obtained from isatin, various α-amino acids and dipolarophiles is considered. This structural fragment plays an important role in biological processes, exhibiting antitumor activity, anti-HIV activity, anti-inflammatory, and, in some cases, analgesic effects.

A 2016 review addressed the development of 1,3-dipolar cycloaddition over the past decade, examining the reactions of various dipoles, including azomethine ylides, with a wide range of dipolarophiles [22]. In reviews [23–25], various methods for the generation of azomethine ylides were discussed in detail, for example, the importance of the formation of azomethine ylides from amino esters, amino acids, imine derivatives, aziridines or other substrates was emphasized. A review [26] demonstrates metal-catalyzed as well as metal-free asymmetric and racemic transformations of imino ethers upon interaction with various dipolarophiles. A review [27] published in 2025 focused on the double cycloaddition of azomethine ylides derived from amino esters or amino acids, which is of great value for the synthesis of complex polyheterocycles.

Currently, the (3 + 2) cycloaddition reactions of azomethine ylides with dipolarophiles having electron-withdrawing substituents have been widely studied; acrylates, vinyl sulfones, maleimides, β-nitrostyrenes, fumarates, and maleates have been used most frequently [28–29]. Reactions of aromatic and heteroaromatic olefins containing both electron-donating and electron-withdrawing groups in the aromatic ring, as well as heterosubstituted alkenes and polyenes, are also known [30–32]. The review [33] examines the reactions of 1,3-dipolar cycloaddition of cyclopropenes with various dipoles, such as diazo compounds, azides, azomethine imines, nitrones, azomethine ylides, carbonyl ylides, etc. At the same time, (3 + 2) cycloaddition involving non-activated alkenes of various structures has not yet been sufficiently studied [3]. A review article [34] examines 1,3-dipolar cycloaddition reactions occurring under organocatalytic conditions. Zhao's and Bayat's works provide a detailed discussion of multicomponent one-pot (3 + 2) cycloaddition reactions involving azomethine ylides from isatins and amino acids [21,35].

The main feature of this review is that we have attempted to consider all currently known systems for generating azomethine ylides based on carbonyl compounds and amino acid derivatives (or amines) and to demonstrate their synthetic potential using a number of examples. The main strategies for synthesizing pyrrolidine derivatives via asymmetric and non-asymmetric 1,3-dipolar cycloaddition of azomethine ylides derived from cyclic and acyclic amino acids or amino esters with various carbonyl compounds were reviewed. Rare methods for preparing azomethine ylides using pyridylimines and α-silylimine were described. Electrophilic alkenes, styrene derivatives, and new, previously undescribed unsaturated substrates were frequently used as dipolarophiles. The contribution of our research group to the development of this method is also presented.

## Review

### Imine derivatives as precursors of azomethine ylides

#### Azomethine ylides based on iminoesters

**Scheme 2:**
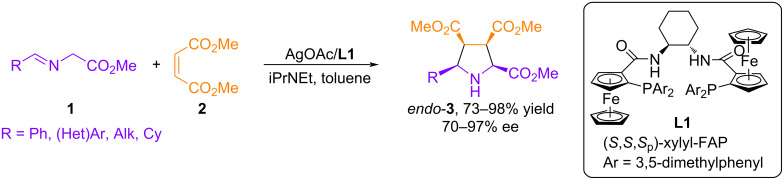
(3 + 2) Cycloaddition of iminoesters to dimethylmaleate.

In 2002, Zhang and co-workers described a highly enantioselective Ag(I)-catalyzed (3 + 2) cycloaddition of azomethine ylides from α-(arylimino)esters using AgOAc as the catalytic precursor and chiral bis-ferrocenyl amide phosphine (FAP) (**L1**) as ligand [36] (Scheme 2). The obtained results demonstrated the high efficiency of the Ag(I)-FAP catalytic system for this transformation. In particular, most α-(arylimino)esters yielded cycloaddition products **3** in excellent yields and with high diastereoselectivity and enantioselectivity (up to 97% ee). However, α-(alkylimino)esters were less reactive, requiring longer reaction times and yielding products with slightly lower enantioselectivity (up to 81% ee).

**Scheme 3:**
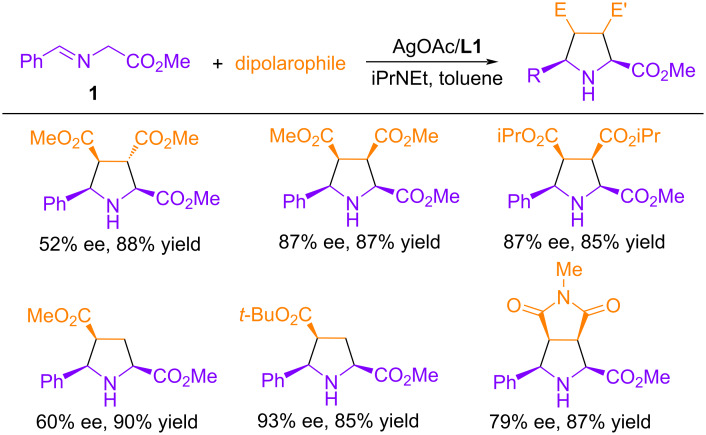
Cycloaddition of **1** with various dipolarophiles catalyzed by Ag(I)-**L1**.

In addition to dimethyl maleate, other dipolarophiles, such as dimethyl fumarate, acrylates, and *N*-methylmaleimide, were also studied in the cycloaddition reaction (Scheme 3). Lower enantioselectivity was observed in these cases, and the most important result was a significant improvement in enantioselectivity upon switching from methyl acrylate to bulk *tert*-butyl acrylate, 60 and 93% ee, respectively.

**Scheme 4:**
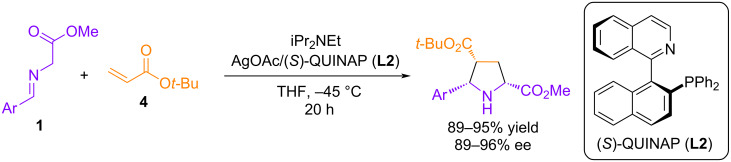
Cycloaddition of **1** with *tert*-butyl acrylate catalyzed by Ag(I)-**L2**.

In 2003, Schreiber et al. reported an efficient silver(I) acetate/QUINAP (**L2**) catalytic system for the (3 + 2) cycloaddition of azomethine ylides to unsaturated carboxylic acid esters [37]. The reaction with *tert*-butyl acrylate showed excellent levels of diastereoselectivity (>20:1) and enantioselectivity (89–96% ee) regardless of the electronic and steric properties of the aromatic ring in the α-(arylimino)ester (Scheme 4). However, when using other dipolarophiles, such as dimethyl maleate, *tert*-butyl crotonate, and *tert*-butyl cinnamate, a noticeable decrease in enantioselectivity was observed.

**Scheme 5:**
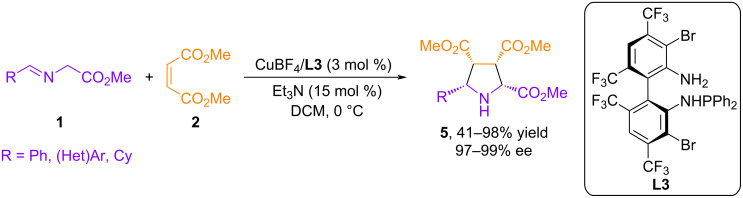
Cycloaddition of **1** with dimethyl maleate catalyzed by Cu(I)-**L3**.

In a similar study, Wang et al. reported the CuI/TF-BiphamPhos (**L3**) complex, a new and highly efficient catalyst for the asymmetric 1,3-dipolar cycloaddition reaction [38]. The authors noted excellent reactivity, selectivity, and a wide range of structural variants for various azomethine ylides derived from amino esters, leading to the formation of the corresponding cycloadducts **5** with exceptionally high enantioselectivity (Scheme 5).

**Scheme 6:**
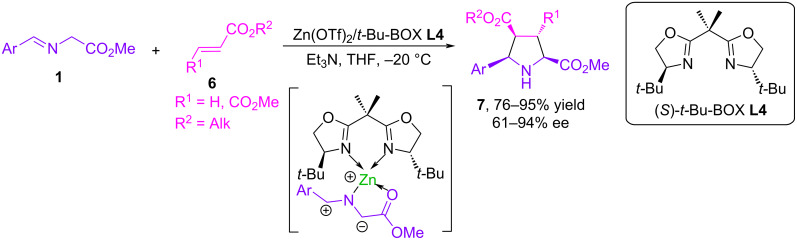
Cycloaddition of **1** with alkenes catalyzed by Zn(II)-*t-*Bu-BOX **(L4)**.

In a classic paper published in 2002, Jørgensen investigated the catalytic asymmetric 1,3-dipolar cycloaddition of azomethine ylides from *N*-(arylmethylidene)glycinates with unsaturated substrates. The reactions were successfully catalyzed by the Zn(II)-*t*-Bu-BOX complex (**L4**) and produced diastereomerically pure, highly functionalized pyrrolidines in high yields and enantiomeric excesses of up to 94% (Scheme 6) [39]. Based on the data obtained, the authors proposed a model for the intermediate complex in 1,3-dipolar cycloaddition reactions. This intermediate consists of an azomethine ylide coordinated to a Zn(II)-*t*-Bu-BOX catalyst and is an 18-electron complex with a tetrahedral arrangement of ligands around the zinc center. Excellent results for yields, diastereo- and enantioselectivities of analogous (3 + 2) cycloaddition reactions were achieved using new chiral catalytic systems, in particular, Zn(II)-imidazolinyl-[2.2]paracyclophanol (UCDImphanol) ligand [40] and Zn(II)-ferrocenyl-substituted aziridino alcohol ligand [41].

**Scheme 7:**
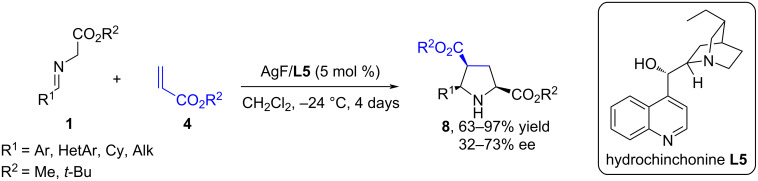
(3 + 2) Cycloaddition of iminoesters to acrylates.

Later in 2005, the Jørgensen group presented an asymmetric strategy for the 1,3-dipolar cycloaddition of azomethine ylides based on iminoesters **1** with alkyl acrylates **4** using a metal salt of AgF with the chiral base hydrochinchonine **L5** as a catalyst system (Scheme 7) [42]. The cycloaddition of iminoesters **1**, obtained from glycinates and aromatic or aliphatic aldehydes, to alkyl acrylates **2** proceeds with high *endo*-diastereoselectivity and moderate enantioselectivity. However, most pyrrolidine derivatives **8**, obtained from *tert*-butyl acrylate, are solids that can be enantiomerically enriched by recrystallization.

**Scheme 8:**
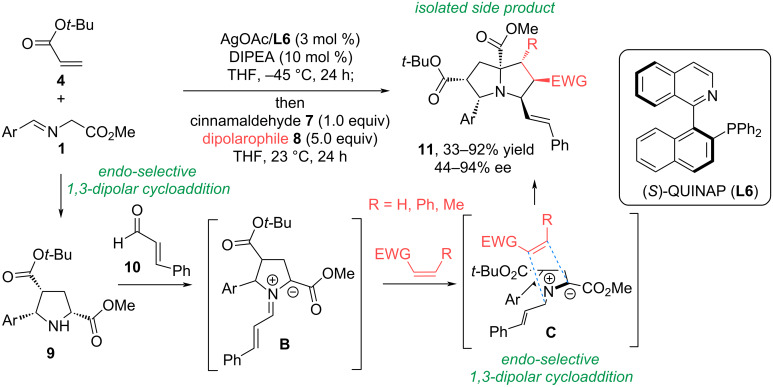
Catalytic double (3 + 2) cycloaddition to form pyrrolizidine derivatives.

In [43], a silver-catalyzed asymmetric double 1,3-dipolar cycloaddition reaction was developed that provides highly substituted, enantioenriched pyrrolizidines **9** containing up to six stereocenters (Scheme 8). It is assumed that the *endo*-selective (3 + 2) cycloaddition of the azomethine ylide obtained from iminoester **1** with *tert*-butyl acrylic acid ester **4** occurs first. This is followed by condensation of pyrrolidine **9** with cinnamic aldehyde (**10**) to form azomethine ylide **B**, which enters into a second diastereoselective 1,3-DC with various electrophilic alkenes. The authors used acrylic acid esters, vinylphenyl sulfone, cinnamic, croton, and methacrylic aldehydes, and β-nitrostyrene as dipolarophiles. Depending on the dipolarophile used, pyrrolizidines **11** were obtained with an enantiomeric excess of up to 94% and yields of up to 92%.

**Scheme 9:**
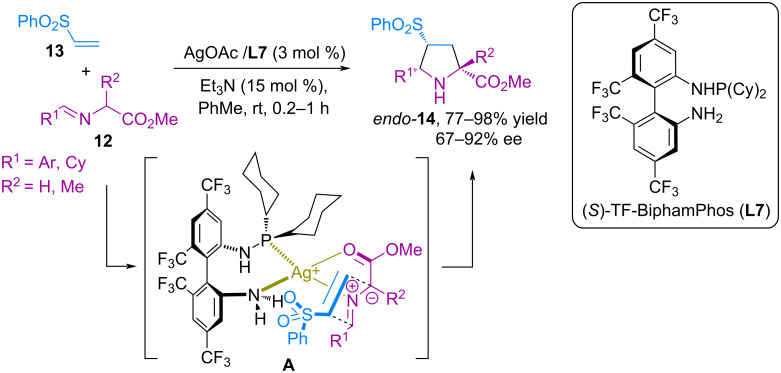
(3 + 2) Cycloaddition of iminoethers to vinyl phenyl sulfone.

In 2009, Wang and co-workers reported the catalytic *endo*-selective cycloaddition of azomethine ylides based on glycine or alanine methyl ester imines **12** with vinyl phenyl sulfone **13** catalyzed by a chiral silver complex (Scheme 3) [44]. It was found that iminoethers **12** are capable of reacting smoothly with vinylphenylsulfone **13**, forming the corresponding *endo*-cycloadducts **14** in high yields (77–98%) and good enantioselectivity (67–92% ee). The in situ-generated azomethine ylide is coordinated to the metal center and oriented in transition state **A** due to the spatial repulsion between the aryl or cyclohexyl group in the ylide and the cyclohexyl fragments of ligand **L7**, while its significant size effectively blocks the approach of the dipolarophile to the *Re* surface of the ylide and forms the *endo*-product as a result of exposure to the *Si* surface (Scheme 9).

**Scheme 10:**
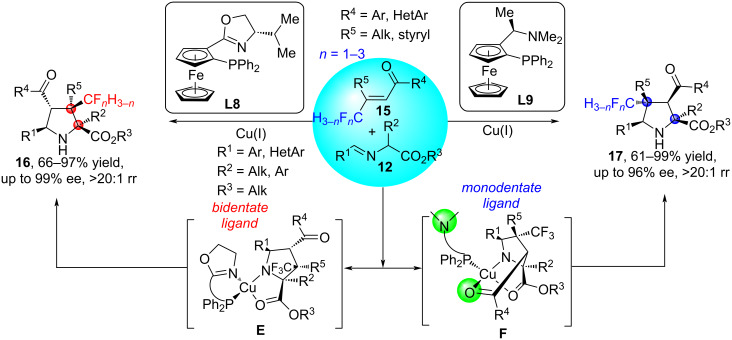
Regiodivergent and enantioselective synthesis of pyrrolidines **16** and **17**.

In [45], the Cu(I)-catalyzed enantioselective and regiodivergent asymmetric (3 + 2) cycloaddition of α-substituted iminoesters **12** with β-fluoromethyl-β,β-disubstituted enones **15** was studied, resulting in the formation of pyrrolidines **16** and **17** with two adjacent or two discrete quaternary stereocenters (Scheme 10). The regioselectivity of this reaction is controlled by the choice of a suitable chiral ligand. The authors hypothesized that when ligand **L8** is utilized, throughout the entire catalytic cycle both the nitrogen and phosphorus atoms of the ligand maintain their coordination to copper. The final product **16** is formed as a consequence of electron redistribution within the metal-ligand assembly, coupled with steric constraints and π–π interactions occurring in transition state **E** between the aromatic rings of the enone and iminoester substrates. It has been also demonstrated that in this particular (3 + 2) cycloaddition reaction ligand **L9** acts as a pseudo-bidentate ligand. The formation of the enone Cu−O bond and the dissociation of the amine nitrogen of **L9** from the Cu(I) center in the transition state **F** leads to a change in regioselectivity and the formation of cycloadduct **17** [46]. A similar catalytic system was successfully used for the asymmetric (3 + 2) cycloaddition of azomethine ylides with trisubstituted cyclopropenes, resulting in a variety of complex 3-azabicyclo[3.1.0]hexane derivatives as a single isomer in excellent yields (up to 99%) and enantioselectivities (97–99% ee) [46].

**Scheme 11:**
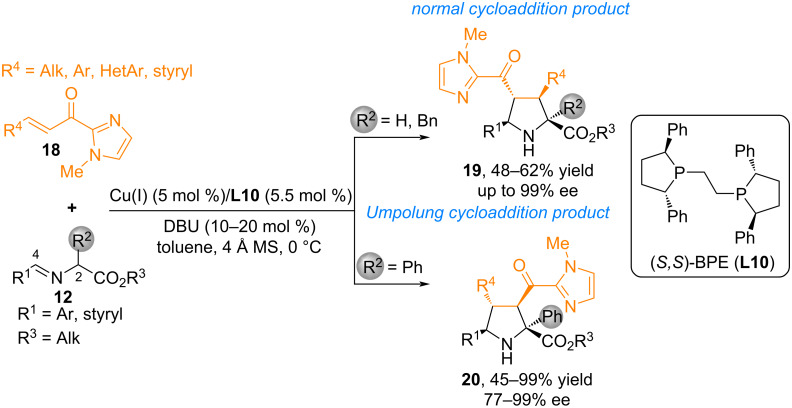
Substrate-controlled regioreversible "normal" and "incomplete" 1,3-dipolar cycloaddition.

In [47], Singh and co-workers reported an enantioselective (3 + 2) cycloaddition reaction of iminoesters **12** with substituted imidazoles **18** catalyzed by Cu(I)/phosphine **L10**, which resulted in the formation of chiral pyrrolidines **19** and **20** with high diastereoselectivity (Scheme 11). The authors attribute the reactivity patterns observed in imino esters reactions, including regioselectivity variations, by differences in electron density distribution between the C^2^ and C^4^ positions following deprotonation. An aryl group located at the α-position (C^2^) greater facilitates the resulting negative charge delocalization, thereby reducing electron density at C^2^ relative to C^4^. Consequently, this stabilized carbanion directs the formation of a product exhibiting reversed polarity (**20**). Conversely, when no α-aryl substitution is present, increased electron density remains at the C^2^ position, favoring the typical (3 + 2) cycloaddition pathway leading to product **19**.

**Scheme 12:**
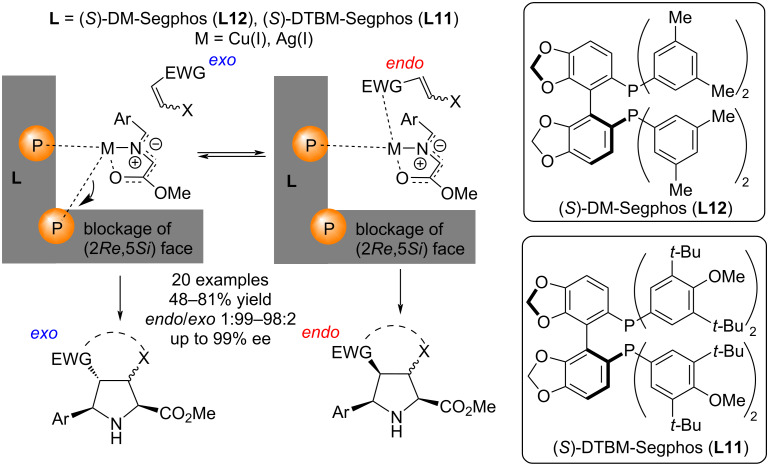
Enantioselective synthesis of *exo*-/*endo*-pyrrolidines.

In [48], the authors studied the reason for the complete change in diastereoselectivity in the catalytic (3 + 2) cycloaddition between iminoethers and electrophilic alkenes occurring using chiral metal complexes based on copper(I) and silver(I) salts and ligands (*S*)-DM-Segphos (**L12**) or (*S*)-DTBM-Segphos (**L11**) (Scheme 12). The reactions involved various dipolarophiles such as acrylates, maleimides, alkenyl sulfones, β-nitrostyrene, chalcone, acrylonitrile, *N*,*N*-dimethylacrylamide, and *N*-acryloyloxazolidin-2-one. Using computational density functional theory (DFT), the authors concluded that the diastereoselectivity of the reaction is influenced by the size of the ligand, the presence or absence of interaction between the metal and the electron-withdrawing group of the dipolarophile, and the coordination properties of the metal, for example, the possibility of changing the coordination sphere of copper(I) from bidentate to monodentate, which does not occur with the Ag(I) atom, which has stronger bonds with the ligands [42].

**Scheme 13:**
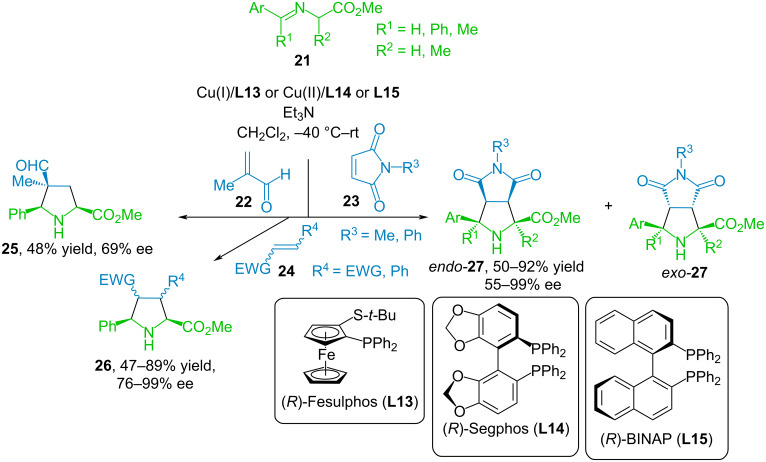
(3 + 2) Cycloaddition of iminoethers **21** to dipolarophiles **22**–**24**.

In [49–50], a variety of dipolarophiles **22**–**24** containing electron-withdrawing substituents was demonstrated, which can participate in the 1,3-dipolar cycloaddition catalyzed by chiral copper complexes Cu(I) or Cu(II) with arylimines of glycine or alanine methyl esters **21** (Scheme 13). In these reactions, *N*-substituted maleimides, acyclic deactivated alkenes such as dimethyl maleate, dimethyl fumarate and fumarodinitrile, and monoactivated alkenes such as methyl acrylate, β-nitrostyrene and methacrolein have shown high activity.

**Scheme 14:**
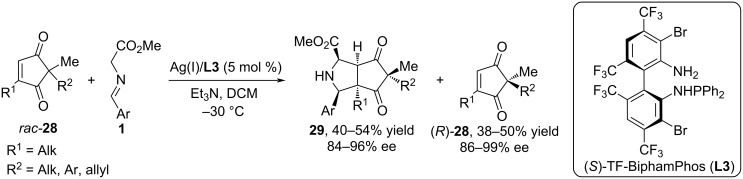
Synthesis of bicyclic pyrrolidines **29** from cyclopentene-1,3-diones.

In [51–52], the authors demonstrated the possibility of using racemic cyclopentene-1,3-diones **28** as dipolarophiles in 1,3-dipolar cycloaddition reactions using the chiral complex Ag(I)/TF-BiphamPhos (**L3**) (Scheme 14). The authors used various substituted diones **28** containing aromatic and aliphatic substituents, for example, silyloxy, acetoxy, benzyloxy, etc. It was established that the reaction of glycine methyl ester arylimines **1** with racemic cyclopentene-1,3-diones **28** leads to bicyclic pyrrolidines **29** and enantiomerically enriched cyclopentenediones (*R*)-**28** in moderate yields. The Ag(I)/**L3** catalytic system also showed high efficiency in the asymmetric (3 + 2) cycloaddition of iminoethers with various 2-alkylidenecyclopentanones [53]

**Scheme 15:**
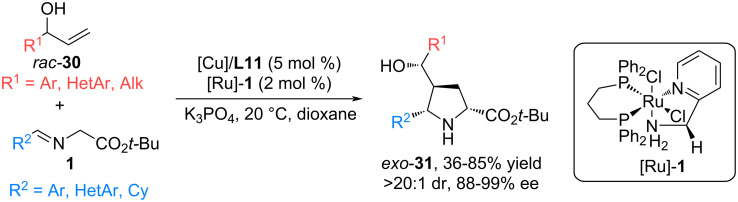
(3 + 2) Cycloaddition of aldimine esters and allyl alcohols using copper-ruthenium catalysis.

In 2025, Wang and co-workers carried out a cascade enantioconvergent reaction between aldimine esters **1** and low-reactivity allylic alcohols **30**, which are activated by oxidative dehydrogenation to form enones, which trigger a 1,3-dipolar cycloaddition via copper–ruthenium catalysis [54] (Scheme 15). Further reductive hydrogenation of ketopyrrolidines proceeds to form hydroxypyrrolidines **31** in high yields and excellent diastereo-/enantioselectivity. The reaction can be used with aldimine esters with various aromatic and aliphatic substituents; vinylaryl carbinols with electron-withdrawing and electron-donating substituents in the phenyl ring have successfully proven themselves as dipolarophiles; replacement of the aryl substituent with heteroaromatic or aliphatic ones also led to acceptable results.

The authors used this methodology to modify peptides and functionalize several natural products. Thus, aldimine dipeptides Gly–Gly, Gly–Ala, as well as iminoesters in combination with natural ʟ-borneol, ʟ-menthol, cholesterol, or lactate scaffolds proved to be suitable precursors of azomethine ylides, allowing the production of functionalized pyrrolidines with potential biological activity [54].

**Scheme 16:**
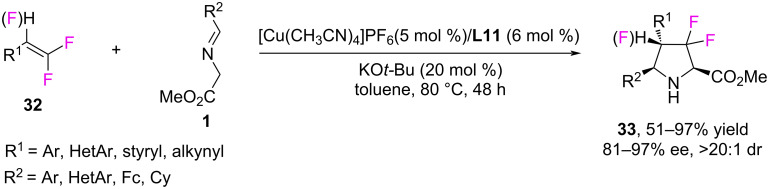
Synthesis of 3,3-difluoro- and 3,3,4-trifluoropyrrolidine derivatives.

Currently, organofluorine chemistry is one of the most pressing areas of modern chemistry, since the introduction of fluorine-containing groups into compounds often has a significant impact on their biological activity. In 2022, Wang and Teng developed an efficient method for the preparation of enantioenriched derivatives of 3,3-difluoro- and 3,3,4-trifluoropyrrolidines **33** in yields of up to 97% via Cu(I)-catalyzed enantioselective 1,3-dipolar cycloaddition of azomethine ylides to 1,1-difluoro- and 1,1,2-trifluorostyrenes **32** (Scheme 16) [30]. The best results were achieved using [Cu(CH_3_CN)_4_]PF_6_/(*S*)-DTBM-Segphos **L11** as the catalytic system.

**Scheme 17:**
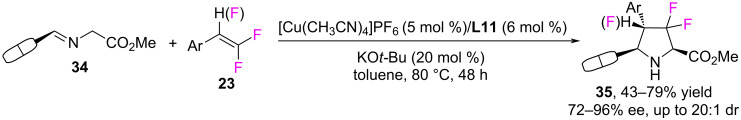
Use of iminoesters from natural compounds and pharmaceuticals for reactions with 1,1-difluoro- and 1,1,2-trifluorostyrenes.

The authors extended the scope of application of this method to various aryl-substituted iminoesters containing furyl, thienyl, indolyl, triazolyl, and ferrocenyl substituents. Various aryl-substituted *gem*-difluorostyrenes, not only with electron-withdrawing but also with electron-donating and neutral substituents in the aromatic ring, can also participate in this reaction, since cycloaddition is facilitated by the electron-withdrawing inductive effect of fluorine atoms. In addition to *gem*-difluorostyrenes, 1,1,2-trifluorostyrenes can be used in the synthesis, which leads to the formation of 3,3,4-trifluoropyrrolidines in moderate yields and high diastereoselectivity [30]. In addition to the above, the authors demonstrated the broad applicability of this method using iminoesters **34** derived from natural compounds or currently available synthetic drugs (Scheme 17). Iminoesters containing molecules of cholesterol, androsterone, indomethacin, pitavastatin, menthol, as well as fructose and glucose, were introduced into the reaction.

**Scheme 18:**
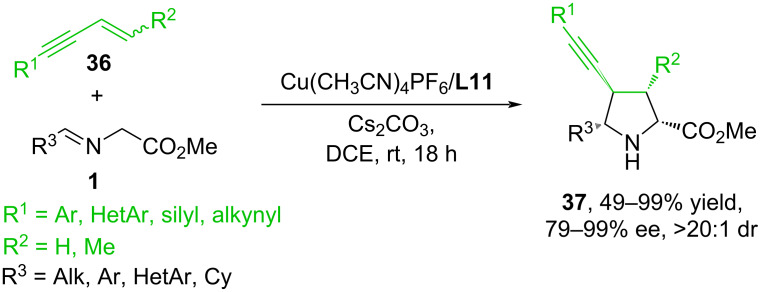
Reaction of iminoesters with 1,3-enynes.

**Scheme 19:**
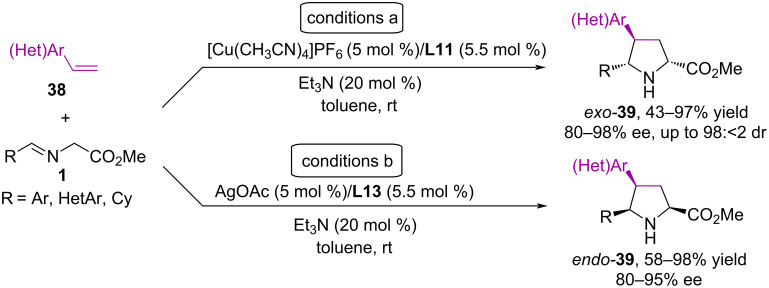
Synthesis of pyrrolidines from iminoesters and vinyl(hetero)arenes.

In [55], copper(I)-catalyzed asymmetric 1,3-dipolar cycloaddition of iminoesters **1** and 1,3-enynes **36** is described, which provides a series of chiral polysubstituted pyrrolidines **37** with high regio-, diastereo-, and enantioselectivities (Scheme 18). It is noted that the presence of a conjugated triple bond leads to weak activation of the olefin group and, thus, somewhat simplifies the cycloaddition reaction. The introduction of aryl and alkynyl substituents at the C4 position of the dipolarophile increases the reactivity of the multiple bond due to double activation by conjugated groups. In 2016, a study was published [56] in which vinyl(hetero)arenes **16** were investigated as dipolarophiles in Cu^I^- and Ag^I^-catalyzed 1,3-dipolar cycloaddition reactions with various imines **1** (Scheme 19).

**Scheme 20:**
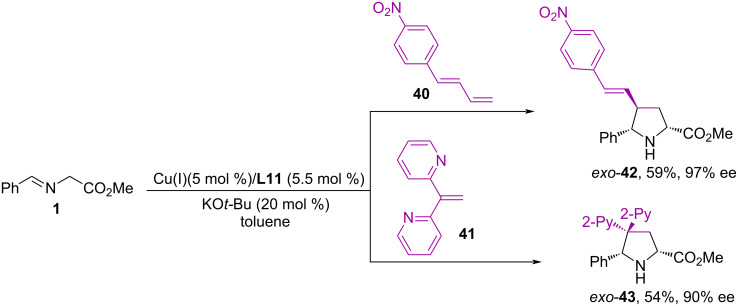
Synthesis of *exo*-pyrrolidines **42** and **43**.

It is noteworthy that when using the [Cu(CH_3_CN)4]PF_6_/(*R*)-DTBM-Segphos **L11** catalytic system, *exo*-pyrrolidines **39** are formed with >98% ee and almost complete diastereoselectivity, while the AgOAc/(*R*)-Fesulphos **L13** catalytic system gives an inversion of diastereoselectivity and the formation of predominantly *endo*-adduct **39** with up to 95% ee (Scheme 19). The authors used various substituted styrenes in the reactions, as well as vinyl heteroarenes containing 2-thiazolyl, 2-quinolyl, 2-pyridyl, and 4-pyridyl fragments. It is worth noting that 1-(4-nitrophenyl)-1,3-butadiene (**40**) and 1,1-bis(2-pyridyl)ethylene (**41**) also proved to be suitable substrates in this reaction (Scheme 20). It was demonstrated by computational methods (DFT) that electronic effects within the dipolarophile can lead to a change in the mechanism and to an effective electrophilicity polarization toward the terminal carbon atom. This finding strongly supports the suitability of moderately activated olefins as dipolarophiles for the catalyzed asymmetric cycloaddition reactions involving azomethine ylides [56].

**Scheme 21:**
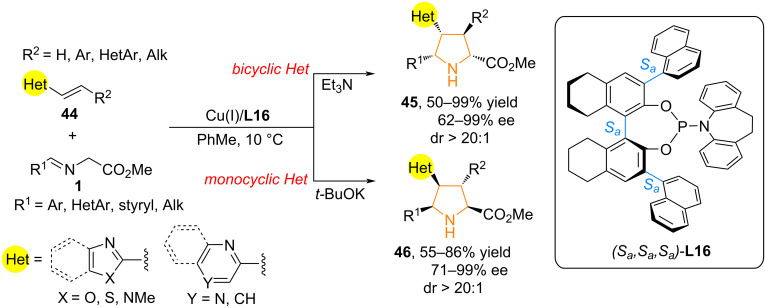
Enantioselective synthesis of heteroarylpyrrolidines **45** and **46**.

In 2021, Wang and co-workers developed a novel catalytic system based on Cu(I) and the bulky phosphoramidite ligand **L16** with triple homoaxial chirality, allowing β-substituted alkenylheteroarenes **44**, which lack a strong electron-withdrawing substituent in heteroarenes, to act as effective dipolarophiles in 1,3-dipolar cycloaddition reactions. This resulted in chiral compounds **45** and **46** containing two biologically important moieties: a heteroarene and a pyrrolidine (Scheme 21) [31].

This synthesis method is applicable to a wide range of substrates: imino esters containing aryl, alkyl, and heteroaryl substituents; various β-aryl- and alkyl-substituted alkenyl heteroarenes, such as benzoxazole, benzothiazole, 1-methyl-1*H*-benzo[*d*]imidazole, dihydrooxazole, and isoquinoline, as well as monocyclic heteroarenes, including oxazole, thiazole, imidazole, pyridine, and pyrazine, can be used as dipolarophiles. The pyrrolidines obtained during the synthesis exhibit exceptional diastereoselectivity (>20:1 dr) and excellent enantioselectivity (up to 99%). The authors suggested that the strong steric repulsion between the 1-naphthyl group of ligand **L16** and the bicyclic substituent in the dipolarophile is a key factor in controlling enantioselectivity. Replacing the bicyclic heteroarene with a monocyclic one reduces the steric burden and inverse enantioselectivity is observed [31].

**Scheme 22:**
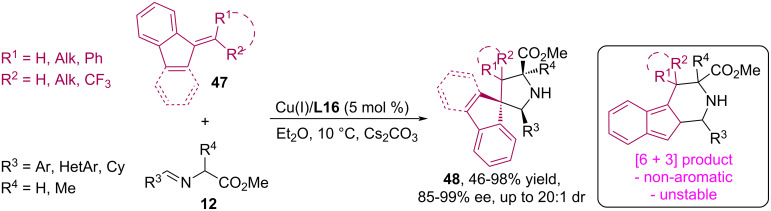
Catalytic reaction of (3 + 2) cycloaddition of imines **12** to benzofulvenes **47**.

Today, the reactions of [6 + 3] cycloaddition of imines to fulvenes, catalyzed by chiral metal complexes, are well known, during which piperidine derivatives are formed [57–58]. In 2023, Wang's research group developed a (3 + 2) cycloaddition strategy of azomethine ylides from iminoethers **12** and benzofulvenes **47**, in which benzofulvenes without electron-withdrawing substituents can act as active 2π-dipolarophiles for the efficient synthesis of a series of polysubstituted enantioenriched pyrrolidine derivatives **48** possessing a spiroindene molecular structure (Scheme 22) [59].

Based on the results of DFT calculations, it was suggested that the [6 + 3] cycloaddition product for benzofulvene **47** loses aromatic stability and exhibits high steric hindrance, which makes the [6 + 3] cycloadduct extremely unstable and also increases its reaction barrier (29.6 kcal mol^−1^). At the same time, the formation of the (3 + 2) cycloaddition product **48** is thermodynamically more favorable, which is the main factor controlling chemoselectivity. It is noteworthy that carrying out the reaction in the same catalytic system with the simplest fulvene, 6,6-dimethylfulvene, promotes [6 + 3] cycloaddition [59].

**Scheme 23:**
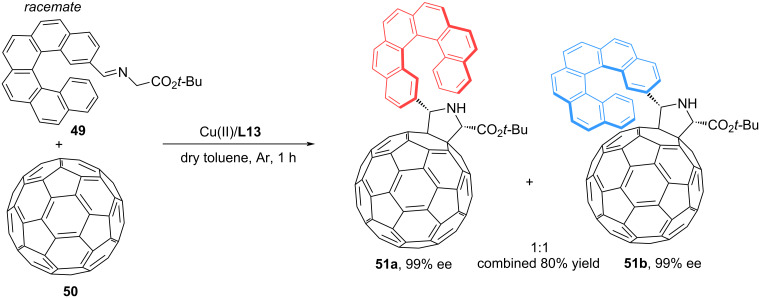
Fullerene as a dipolarophile in (3 + 2) cycloaddition reactions.

In [60], Martín and colleagues investigated the possibility of using C60 fullerene as a dipolarophile in 1,3-dipolar cycloaddition reactions. Due to their unique chemical properties and high versatility, fullerenes are finding increasing application in organic synthesis. In this work, the authors focused on the functionalization of C_60_ with a helicene component via Cu(II)/**L13**-catalyzed enantioselective 1,3-dipolar cycloaddition of racemic 2-hexalicene iminoester **49** to C_60_ fullerene **50** (Scheme 23).

During the reaction, two diastereomeric helicenepyrrolidino[3.4:1.2][60]fullerenes **51a** and **51b** are formed with a good enantiomeric excess. Further separation of the two diastereomers using column chromatography allows one to obtain optically pure products. Thus, fullerene can be used as an effective template for the chiral resolution of racemates [60].

**Scheme 24:**
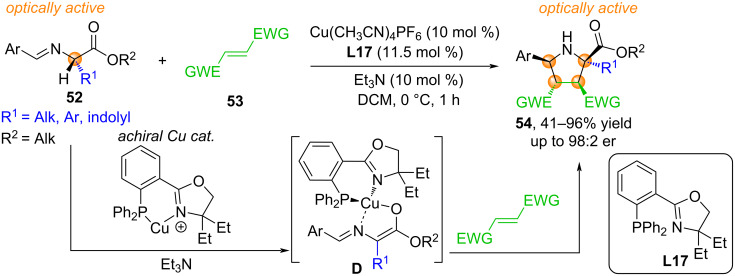
Asymmetric synthesis of optically active tetrasubstituted pyrrolidines **54**.

In 2025, Morisaki and Sato reported an asymmetric synthesis termed “reflexive chirality transfer”, which provides direct access to optically active tetrasubstituted pyrrolidine derivatives **54** from readily available optically pure amino acids without any external chiral additives [4] (Scheme 24). Deprotonation and coordination of optically active imines **52**, obtained from various natural or synthetic α-amino acids, such as ʟ-alanine, ʟ-tryptophan, ʟ-aspartic acid, ʟ-glutamic acid, ʟ-serine, ʟ-methionine, β-naphthylalanine, phenylglycine, 4-iodophenylalanine, on the achiral Cu(I)/**L17** complex leads to the formation of enolates with copper-centered chirality in the form of complex **D**. Next, complex **D** interacts with dipolarophiles **53**, including fumaronitrile, *N*-substituted maleimides, β-nitrostyrene, forming optically active tetrasubstituted pyrrolidines **54** [4].

**Scheme 25:**
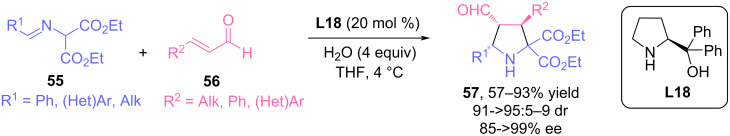
(3 + 2) Cycloaddition reaction of imines **55** and α,β-unsaturated aldehydes.

We would like to separately note the organocatalytic (metal-free) approach to enantioselective syntheses involving azomethine ylides from imino esters. In recent years, thanks to intensive efforts of several research groups, a number of very efficient protocols have been developed to carry out this reaction in an organocatalytic asymmetric version. In particular, Vicario [61–64] reported the first organocatalytic enantioselective (3 + 2) cycloaddition reaction between α,β-unsaturated aldehydes and azomethine ylides. The reaction proceeded with complete regioselectivity and very high diastereo- and enantioselectivity, affording stereoisomerically pure highly functionalized polysubstituted pyrrolidines in excellent yields (Scheme 25).

**Scheme 26:**
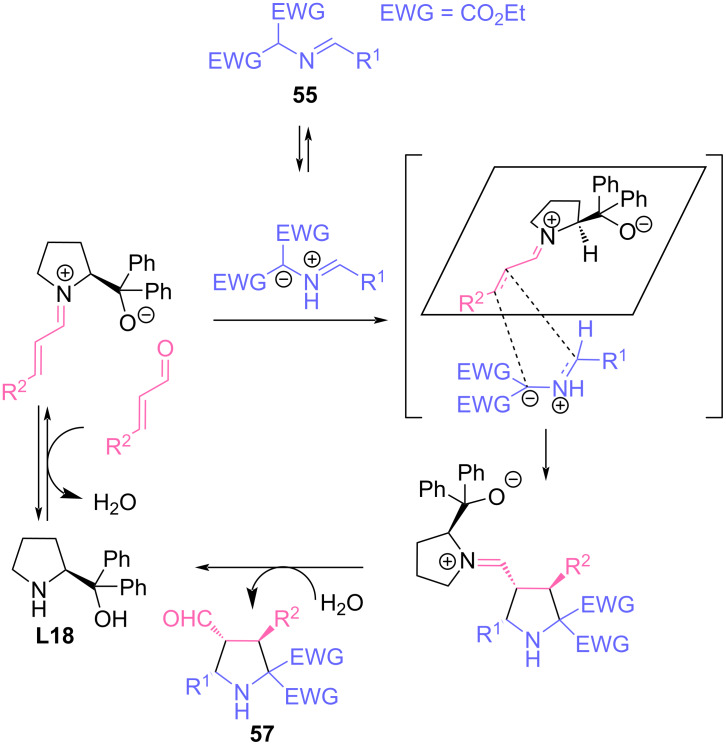
Probable mechanism of enantioselective (3 + 2) cycloaddition of azomethine ylides to α,β-unsaturated aldehydes.

Considering the probable reaction mechanism, the authors suggest that the effective shielding of the *Si*-side of the chiral iminium intermediate by bulky aryl groups leads to a stereoselective *endo* approach of the 1,3-dipole to the sterically less hindered *Re*-side of the intermediate iminium cation (Scheme 26). In this context, it is worth noting a work published in 2011, in which the reaction of analogous azomethine ylides with 2-arylacrylates was catalyzed by chiral binol-based phosphoric acids [65].

**Scheme 27:**
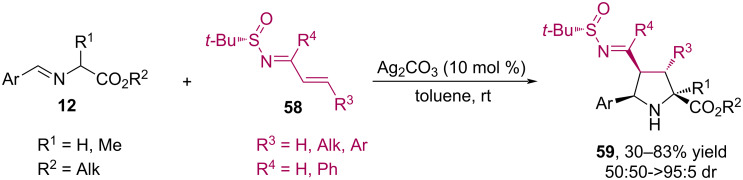
Cycloaddition between iminoesters **12** and sulfinylimines **58**.

In 2023, Retamosa and Sansano carried out a diastereoselective synthesis of polysubstituted pyrrolidines **59** under mild conditions via 1,3-dipolar cycloaddition between *tert*-butylsulfinylimines **58** and iminoesters **12** using Ag_2_CO_3_ as a base (Scheme 27) [66]. Electrophilic dipolarophiles such as sulfinyl imines have been shown to readily undergo (3 + 2) cycloaddition reactions with azomethine ylides generated in situ from the corresponding imino esters, affording pyrrolidines **59** in high yields (up to 83%) and diastereoselectivity (up to 95%). The (*S*)-configuration of the sulfinyl group is capable of inducing the absolute configuration (2*S*,3*R*,4*S*,5*R*) in the final pyrrolidines.

**Scheme 28:**
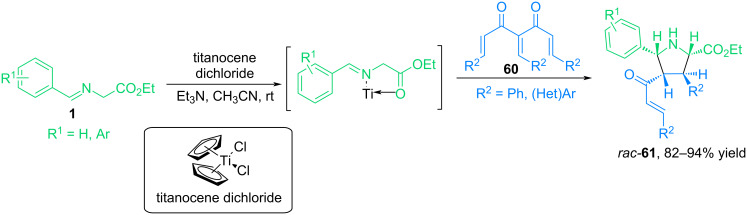
(3 + 2) Cycloaddition between triarylideneacetylacetone and azomethine ylides in the presence of titanocene dichloride.

In the study [67], the 1,3-dipolar cycloaddition reaction of triarylideneacetylacetone derivatives with azomethine ylides derived from glycine ester and aromatic aldehyde, catalyzed by titanocene dichloride, was studied for the first time (Scheme 28). This catalyst demonstrated a number of advantages, including mild reaction conditions, short reaction times, improved yields, and high regio- and stereoselectivity. The cycloaddition proceeded with the formation of *syn*-*endo* cycloadducts **61** and was accompanied by the cleavage of the cinnamoyl group from triarylideneacetylacetone.

In the context of the use of catalytic systems based on metals other than Cu and Ag for the (3 + 2) cycloaddition reactions of N-metalated azomethine ylides, processes catalyzed by Li(I) should be mentioned. The work [68] showed that using Li(I) results in products with different stereoselectivity than those obtained in processes catalyzed by Ag(I). In general, catalytic systems based on lithium salts can be considered as accessible and effective for the preparation of racemic cycloadducts [69–71].

**Scheme 29:**
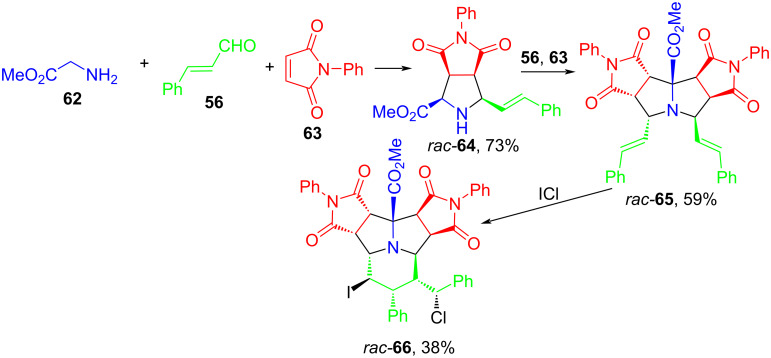
Stereoselective synthesis of decahydropyrrolo[2,1,5-*cd*]indolizine **66**.

Of interest are also non-catalytic reactions of 1,3-dipolar cycloaddition of azomethine ylides, which are formed in situ from the corresponding aldehydes and amino acid esters. In 2011, Shi and Gan proposed a stereoselective approach to the synthesis of a rare heterocyclic system, a fully hydrogenated pyrrolo[2,1,5-cd]indolizine derivative [72] (Scheme 29). The process begins with the 1,3-dipolar cycloaddition of azomethine ylide, obtained from glycine methyl ester (**62**) and cinnamaldehyde (**56**), to *N*-phenylmaleimide (**63**) to form pyrrolidine **64**. Further treatment of **64** with cinnamaldehyde and *N*-phenylmaleimide leads to the second (3 + 2) cycloaddition adduct **65**. Addition of ICl to **65** results in cyclization involving the styrene groups, which is accompanied by the formation of decahydropyrrolo[2,1,5-*cd*]indolizine **66**. The reactions proceed with high stereoselectivity, providing products with eleven chiral carbon atoms in three steps.

**Scheme 30:**
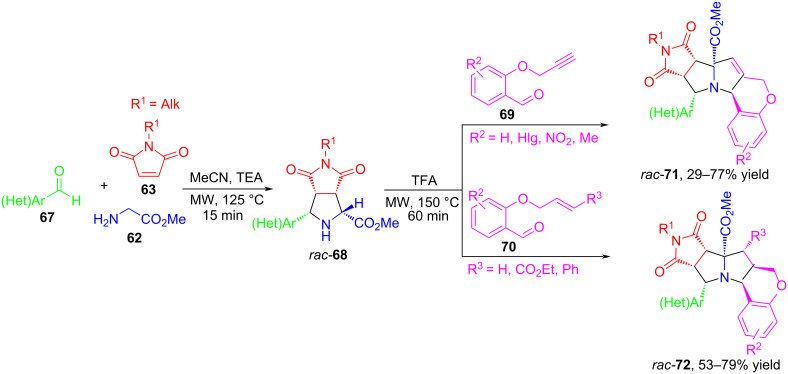
Synthesis of policyclic derivatives **71** and **72**.

In 2018, Zhang and co-workers presented a sequential process based on a combination of inter- and intramolecular (3 + 2) cycloaddition reactions to prepare highly fused heterocyclic systems containing cyclic succinimide, pyrrolidine, pyrrolizidine, and chroman moieties [73] (Scheme 30). The authors showed that the first stage involves intermolecular 1,3-dipolar cycloaddition of azomethine ylides obtained as a result of the interaction of aromatic aldehydes **67** and glycine methyl ester (**62**) to *N*-alkylmaleimides **63**, with the formation of cycloadducts **68**. Next, without isolating the intermediate product **68**, the resulting reaction mixture was used for the intramolecular (3 + 2) cycloaddition step with alkynes **69** and alkenes **70**, which led to the formation of cycloadducts **71** and **72** with high diastereoselectivity.

#### Azomethine ylides based on (2-pyridyl)imines, silylimines and silylamines

**Scheme 31:**
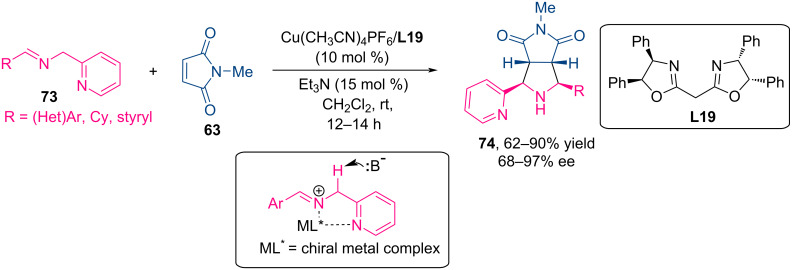
Catalytic аsymmetric (3 + 2) сycloaddition of 2-pyridylimines with *N*-methylmaleimide.

In 2010, Carretero and co-workers demonstrated that *N*-(2-pyridylmethyl)imines **73** are effective precursors of azomethine ylides in catalytic asymmetric (3 + 2) cycloaddition reactions (Scheme 31) [74]. Using Cu(CH_3_CN)_4_PF_6_/bisoxazoline **L19** as a chiral catalyst system, high enantioselectivity (up to 97% ee) and moderate to high *exo*-selectivity were achieved in the reactions with *N*-methylmaleimide. The high efficiency of iminoesters as azomethine precursors is due to the high acidity at the enolizable Cα position and the formation of a five-membered *N,O*-bidentate metalated azomethine. At the same time, a suitable coordinating nitrogen-containing heterocycle, such as a 2-pyridyl group, can also provide sufficient activation via the formation of an *N,N*-bidentate metalated azomethine, which facilitates asymmetric cycloaddition.

**Scheme 32:**
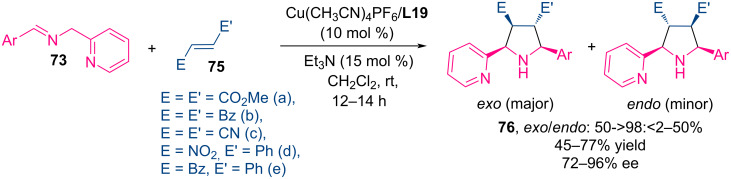
Catalytic аsymmetric (3 + 2) сycloaddition of 2-pyridylimines **1** with other dipolarophiles.

Further studies revealed that reactions of deactivated symmetrical dipolarophiles such as dimethyl fumarate, dibenzoylethylene, and fumarodinitrile proceeded with moderate *exo*-selectivity and good enantioselectivity (72–91% ee) (Scheme 32). Importantly, reactions with unsymmetrically substituted alkenes such as nitroalkenes and enones were completely regioselective, yielding regioisomers in which the activating group is located adjacent to the pyridyl moiety (86–96% ee).

**Scheme 33:**
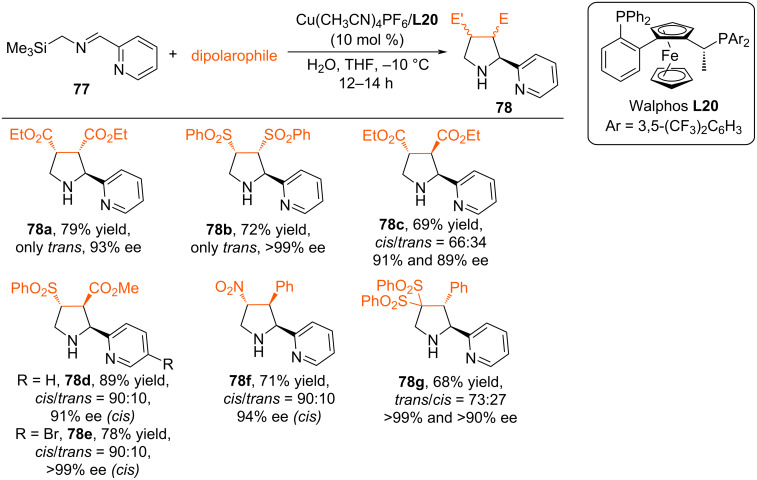
Enantioselective (3 + 2) cycloaddition of silylimine with various dipolarophiles.

The work of Carretero's scientific group has developed effective methodologies for the enantioselective synthesis of complex pyrrolidines, via (3 + 2) cycloaddition of α-silylimines with alkenes [75–76]. In particular, in the work [75] the use of the chiral catalytic system Cu(CH_3_CN)_4_PF_6_/Walphos (**L20**) for the direct enantioselective synthesis of α-heteroarylpyrrolidines via by (3 + 2)-cycloaddition of heteroarylsilylimines with activated alkenes was proposed. The authors demonstrated the potential for cycloaddition of silylimines containing a pyridine substituent with various dipolarophiles (Scheme 33). In all cases, the cycloaddition reactions proceeded with very high diastereoselectivity (*trans*/*cis* >95:<5), providing the *trans* adduct in good yield (47–96%) and high enantioselectivity, regardless of the electronic nature and position of the substituents (82–98% ee).

**Scheme 34:**
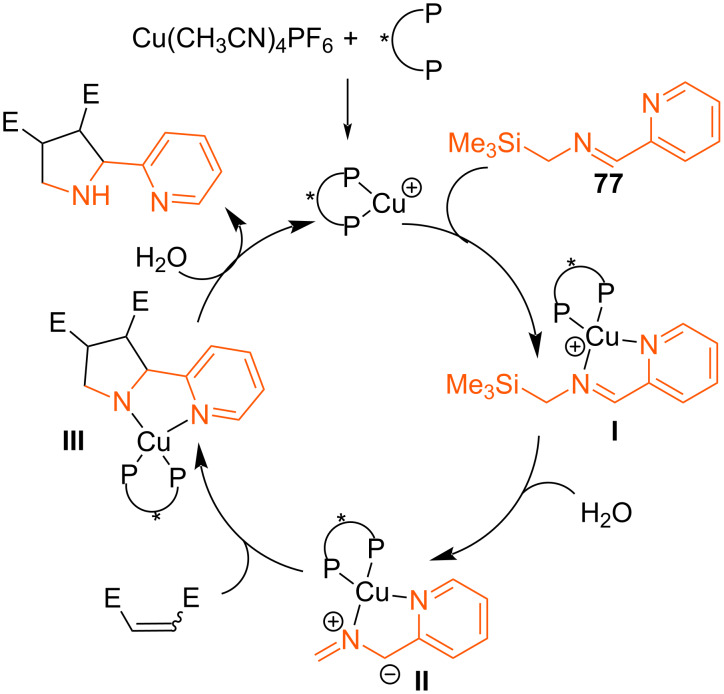
Proposed mechanism of formation of pyrrolidines **78**.

The authors suggest that bidentate coordination of the chiral copper complex with silylimine **77** is crucial for substrate activation, which is presumably accompanied by the formation of *N,N*-complex **I**. Water then promotes the desilylation step, leading to metallodipole **II**, which undergoes cycloaddition with a dipolarophile to form metalated adduct **III**. Final protonation leads to free pyrrolidine (Scheme 34).

**Scheme 35:**
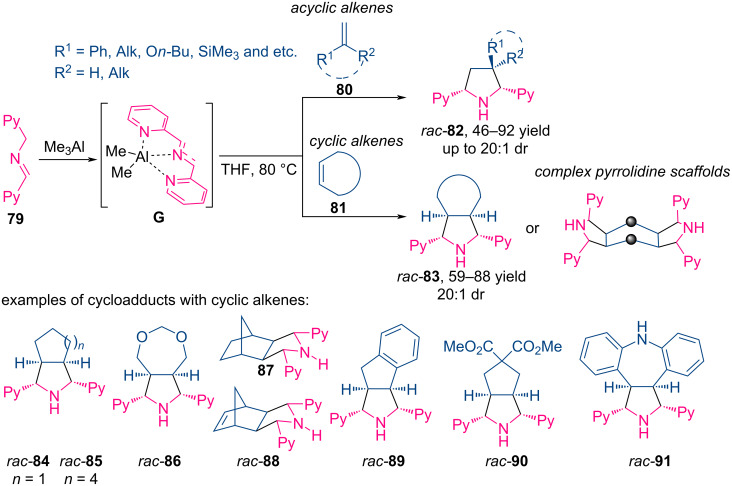
Synthesis of polyheterocyclic pyrrolidines **82**–**91**.

In [3], Mendoza and colleagues developed a universal method for the synthesis of polycyclic pyrrolidines **82**–**91**, which in addition to their use in medicinal chemistry, serve as polydentate *N*-donor ligands. The authors described the trimethylaluminum-catalyzed (3 + 2)-cycloaddition of bis(2-pyridyl)imine **79** with a wide range of acyclic and cyclic olefins **36** (Scheme 35).

This synthesis method is applicable to a wide range of terminally unsubstituted olefins with electron-withdrawing and electron-donating substituents, as well as to non-activated alkenes such as hex-1-ene, styrene, α-methylstyrene, and 2,3-dimethylbutadiene. In addition to the above, various cyclic olefins have shown good performance, in particular derivatives of cyclopentene, cyclooctene, norbornene, norbornanediene, indene, and iminostilbene. In turn, unsymmetrical imines containing aryl, heteroaryl π-deficient (pyrazine) or π-excessive (furan, thiophene, thiazole) substituents can also participate in (3 + 2)-cycloaddition reactions with olefins [3].

**Scheme 36:**
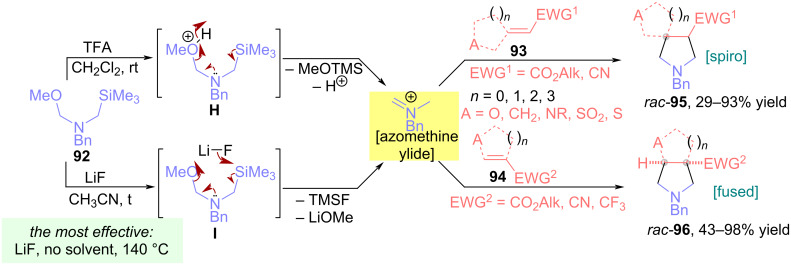
Synthesis of spirocyclic (**95**) and fused (**96**) pyrrolidines.

Above we considered the possibility of generating azomethine ylides from α-silylimines, while a method for forming these dipoles from silylamines is also known. In 2017, Mykhailiuk proposed using *N*-(methoxymethyl)-*N*-(trimethylsilylmethyl)benzylamine (**92**) as an azomethine ylide precursor in (3 + 2)-dipolar cycloaddition reactions with electron-deficient *exo*-cyclic **93** and *endo*-cyclic alkenes **94** (Scheme 36) [77–79]. To generate the azomethine ylide from reagent **92**, TFA in methylene chloride at room temperature or LiF in acetonitrile with heating were used. Notably, this process can proceed solvent-free at 140 °C, which is suitable for less reactive substrates. Various *exo*-cyclic alkenes obtained from cyclic (hetero)aliphatic ketones such as cyclobutanone, azetidinone, thienone, as well as *endo-*(hetero)cyclic alkenes containing oxygen, nitrogen, sulfur or a sulfone group as heteroatoms, reacted with azomethine ylide to form spirocyclic or fused pyrrolidines **95** in yields up to 98%.

The authors suggest that the reaction of silylamine **92** with LiF proceeds via a concerted mechanism: in transition state **I**, the polarized fluorine atom (δ^−^) attacks the silicon atom, while the polarized lithium atom (δ^+^) coordinates with the methoxy group as a Lewis acid, further removal of TMSF and LiOMe leads to the in situ formation of azomethine ylide, which then participates in cycloaddition with alkenes (Scheme 36) [79].

### The decarboxylative route to azomethine ylides

#### Formation of azomethine ylides via decarboxylative condensation of amino acids and aldehydes

Ronald Grigg and colleagues were the first to describe the generation of azomethine ylides by condensation and decarboxylation of primary or secondary α-amino acids with various aldehydes. Early work by Grigg's group investigated intramolecular cycloaddition at terminal double or triple bonds, leading to the formation of fused ring systems [80–81].

**Scheme 37:**
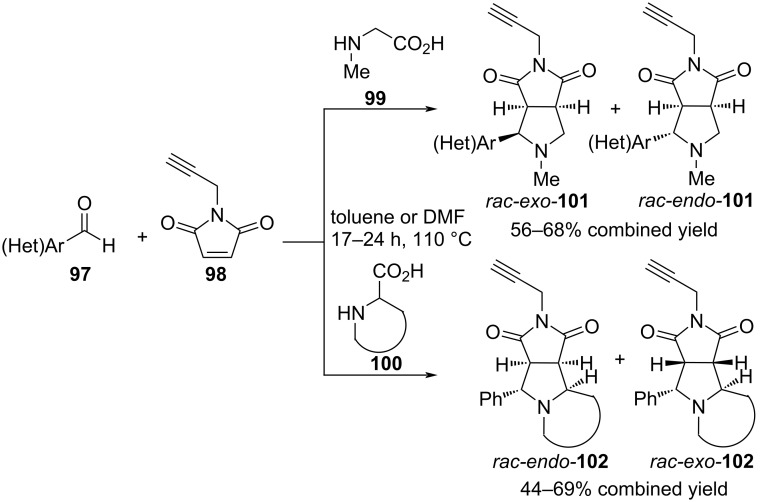
(3 + 2) Cycloaddition involving aromatic aldehydes **97**, *N*-propargylmaleimide (**98**) and α-amino acids **99** and **100**.

In a later work by Grigg's group [82], the cycloaddition of azomethine ylides obtained from aromatic aldehydes **97** and sarcosine (proline, pipecolic acid, or 1,2,3,4-tetrahydroisoquinoline-3-carboxylic acid) **99** (**100**) to *N*-propargylmaleimide (**98**) was described. The reaction yielded a mixture of *endo*- and *exo*-diastereomers **101** and **102** in overall yields of up to 69% (Scheme 37).

**Scheme 38:**
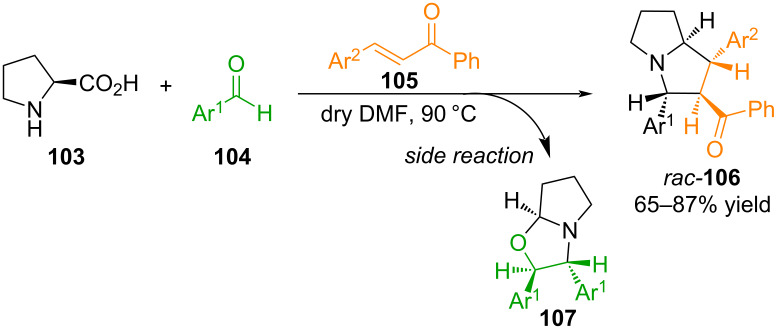
Synthesis of pyrrolizidines **106** and by-product **107**.

In [83], Dhara and co-workers developed a simple strategy for the synthesis of trisubstituted pyrrolizidines **106** via 1,3-dipolar cycloaddition reactions using proline (**103**), arylaldehydes **104**, and electron-deficient dipolarophiles, chalcones **105** (Scheme 38).

The authors suggest that the interaction of proline and arylaldehydes results in the formation in situ of an S-shaped azomethine ylide, which reacts with chalcones with high regio- and stereoselectivity, forming cycloadducts in yields of up to 87%. A side reaction in this case is the cycloaddition of the azomethine ylide to the arylaldehyde to yield substituted oxazolidines **107**. The formation of such a product is due to the possibility of cycloaddition at the carbon–oxygen double bond (C=O) of the aldehyde, which acts as an alternative to the carbon–carbon double bond (C=C) [83].

**Scheme 39:**
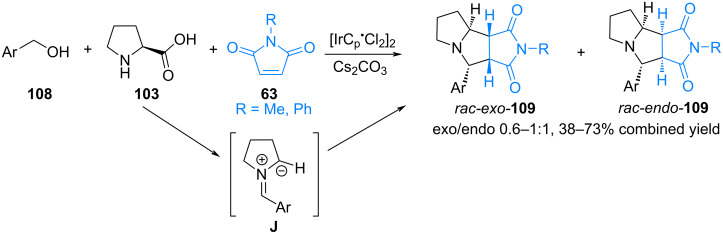
Iridium-catalyzed three-component cascade (3 + 2) cycloaddition.

In 2016, Sridharan reported a one-pot iridium-catalyzed three-component dehydrogenation/1,3-dipolar cycloaddition cascade reaction using benzyl alcohols as aldehyde precursors to prepare fused heterocycles [84]. The authors note that the iridium-catalyzed oxidation of benzyl alcohol **108** to the corresponding aldehyde initially occurs. This is followed by condensation of the aldehyde with proline (**103**) and subsequent decarboxylation to form azomethine ylide **J**, which undergoes a 1,3-dipolar cycloaddition reaction with *N*-substituted maleimides **42d**. This results in a mixture of *endo*/*exo* products **109** in good yields (Scheme 39).

**Scheme 40:**
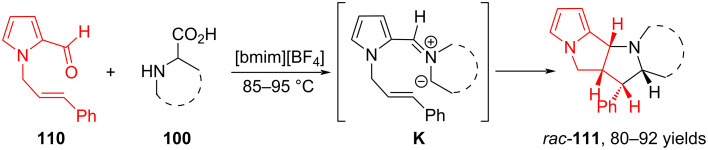
Intramolecular (3 + 2) cycloaddition of *N*-alkenylpyrrole-2-carbaldehyde **110** and α-amino acids.

In [85], Kathiravan and Raghunathan presented an efficient protocol for the synthesis of pyrrolo[2,3-*a*]pyrrolizidine derivatives via intramolecular 1,3-dipolar cycloaddition using [bmim][BF_4_] as a green solvent. When *N*-alkenylpyrrole-2-carbaldehyde **110** reacts with sarcosine or with cyclic secondary amino acids such as proline, thiaproline, pipecolic acid, or isoquinolinic acid **100**, azomethine ylide **K** is formed, which undergoes intramolecular cycloaddition at the multiple bond of the *N*-alkenylpyrrole moiety. As a result, the corresponding derivatives of pyrrolidine, pyrrolizidine, indolizidine and isoquinoline **111** are formed in yields from 80 to 92% (Scheme 40).

**Scheme 41:**
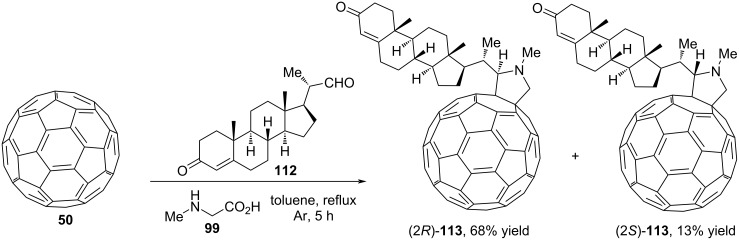
Three-component (3 + 2) cycloaddition involving fullerene.

It is known that when C_60_ is functionalized using fragments of natural molecules such as steroids, peptides or sugars, its solubility in water improves, thereby increasing the possibility of potential biological activity. In 2020, Martín and colleagues investigated fullerene **50** as a dipolarophile in 1,3-dipolar cycloaddition with azomethine ylide based on formyl steroid **112** and sarcosine **99** (Scheme 41) [86]. The synthesis yielded *N*-methyl-2-substituted pyrrolidino[3,4:1,2][60]fullerenes (2*R*/2S)-**113** as a mixture of two diastereomers.

#### Azomethine ylides based on cyclic ketones

**Scheme 42:**
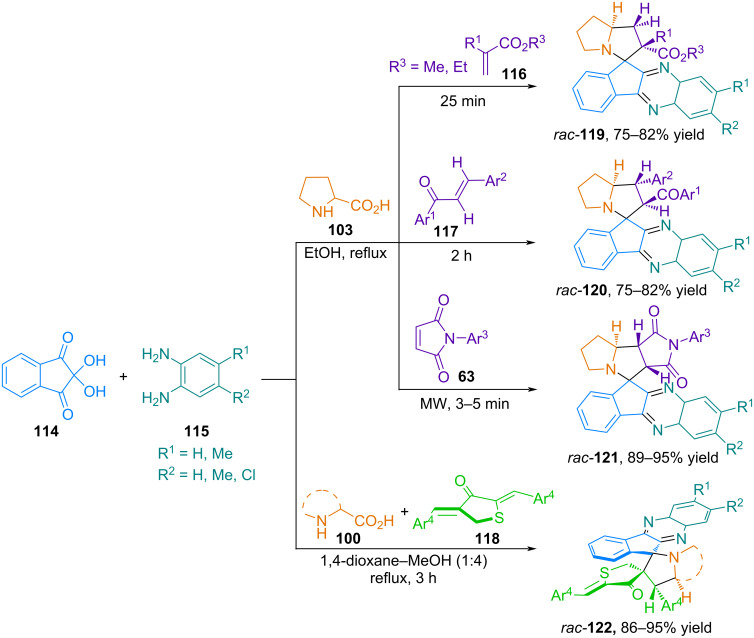
Four-component stereoselective one-pot synthesis of spiro-cycloadducts **119**–**122**.

**Azomethine ylides from ninhydrin.** In [87–90], four-component one-pot reactions of 1,3-dipolar cycloaddition of ninhydrin (**114**), phenylenediamines**115**, α-amino acids, and various dipolarophiles such as alkyl acrylates **116**, chalcones **117**, *N*-arylmaleimides **63**, and arylidene dihydrothiophenones **118** were investigated. The process proceeds stereoselectively with the formation of spiro-fused heterocycles **116**–**122** in high yields (Scheme 42).

When examining the reaction mechanism, the authors proposed that the first step involves the condensation of phenylenediamine and ninhydrin to form indenoquinoxalin-11-one, which then reacts with proline to yield azomethine ylide. The latter subsequently undergoes cycloaddition reactions with various dipolarophiles to stereoselectively obtain spiropyrrolidines. It is noteworthy that the resulting products have three or four (relative to nitrogen) chiral centers, but only one diastereomer is formed during their synthesis, due to the fixed dipole configuration and the structure of the transition state [88].

**Scheme 43:**
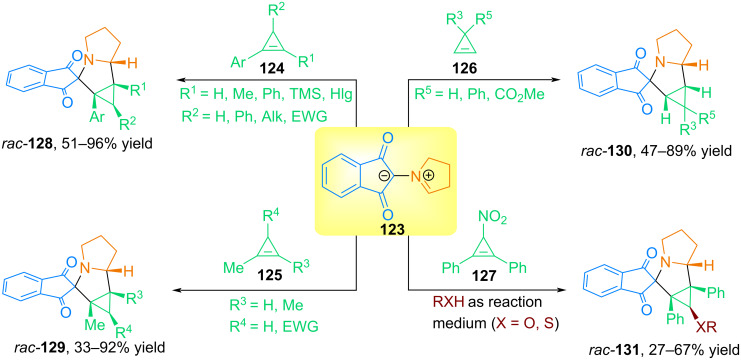
Reactions of azomethine ylide **123** with cyclopropenes.

In [1], we developed an efficient protocol for the diastereo- and regioselective synthesis of spiro[cyclopropa[*a*]pyrrolizine-2,2'-indenes] **128**–**131** using 1,3-dipolar cycloaddition reactions of stable azomethine ylide **123**, obtained in situ by decarboxylating condensation of ninhydrin and ʟ-proline, with a wide range of cyclopropenes, including gaseous and unstable ones (Scheme 43).

In this reaction, symmetrically and asymmetrically substituted cyclopropenes at the multiple bond with various electron-donating and electron-withdrawing substituents at C^3^ were used, resulting in spiro-pyrrolizidines with yields of up to 96%. It should be noted that azomethine ylide **123** can be an effective trap for gaseous cyclopropene and 1-methylcyclopropene, as well as unstable 1-phenylcyclopropene.

The authors also succeeded in carrying out the reaction of **123** with 3-nitro-1,2-diphenylcyclopropene **127** in primary, secondary, and tertiary alcohols, as well as using certain thiols. The authors suggest that the reactions proceed via a stage of heterolytic cleavage of the C–N bond of the starting 3-nitro-1,2-diphenylcyclopropene to form a cyclopropenyl cation, which then reacts with the nucleophile RXH to form the corresponding 3-substituted cyclopropenes. The latter reacts with azomethine ylide **67** to form cycloadducts in moderate yields. Density functional theory (DFT) calculations revealed that the regio- and *endo*-stereoselective formation of products in the observed reactions is due to charge and orbital control [1].

**Scheme 44:**
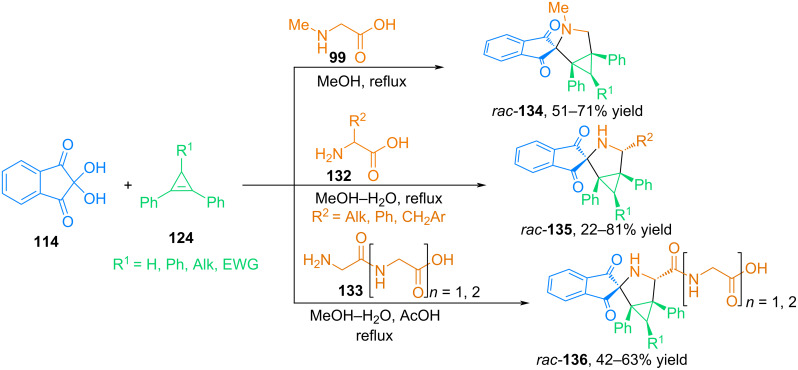
Three-component reactions involving ninhydrin, cyclopropenes and acyclic α-amino acids.

Continuing the study of 1,3-dipolar cycloaddition reactions of ninhydrin-based azomethine ylides with cyclopropene dipolarophiles, our research group examined the interaction of cyclopropenes with azomethine ylides from ninhydrin and acyclic amino acids [91]. The authors carried out three-component reactions involving ninhydrin (**114**), 3-R-1,2-diphenylcyclopropenes **124** and sarcosine (**99**), as well as some primary α-amino acids **132**, such as ʟ-leucine, ʟ-phenylalanine, ʟ-methionine, ʟ-tyrosine, 3,5-diiodo-ʟ-tyrosine, ᴅʟ-phenylglycine (Scheme 44). The authors also carried out cycloaddition reactions of 1,2,3-triphenylcyclopropene and 2,3-diphenylcycloprop-2-enecarboxylic acid with azomethine ylides formed from ninhydrin and peptides **133**, such as Gly–Gly and Gly–Gly–Gly. All reactions proceed under mild conditions to form spiro[3-azabicyclo[3.1.0]hexanes **136** in good yields and excellent diastereoselectivity.

**Scheme 45:**
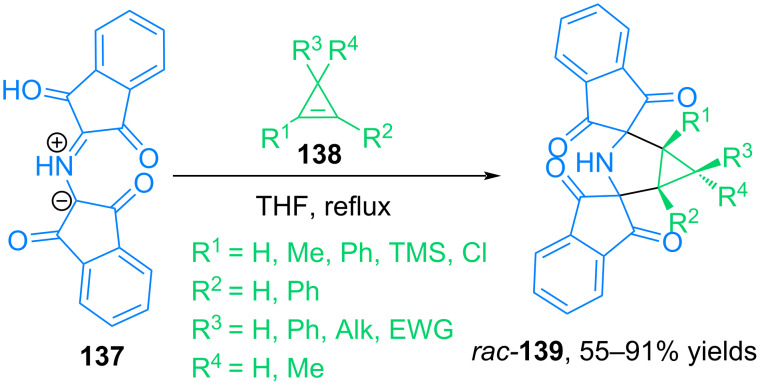
Reaction of cyclopropenes **138** with the *N*-protonated form of Ruhemann purple **137**.

In [92], our group developed a method for the synthesis of bis-spiro[3-azabicyclo[3.1.0]hexanes] **139** from the *N*-protonated form of Ruhemann purple **137** (PRP) and various stable and unstable cyclopropenes **138** (Scheme 45). The study revealed that protonated Ruhemann purple is one of the few known stable azomethine ylides that can be used as an effective trap for various stable and unstable cyclopropenes.

**Azomethine ylides from isatins.** Isatin-based azomethine ylides are important intermediates, as they provide an effective approach to potentially biologically active oxindoles spiro-fused with pyrrolidines. Naturally, the task arises of developing synthetic methods for the organocatalytic 1,3-dipolar cycloaddition of isatin-derived azomethine ylides and exploring their further application in the enantioselective synthesis of spiro-oxindoles. Azomethine ylides from isatin can be formed via two main pathways: the decarboxylative route [93] or 1,2-prototropy [94]. It was on the basis of the second approach to the generation of azomethine ylides, via 1,2-prototropy, that effective organocatalytic enantioselective methods for the preparation of spiro-oxindoles were developed.

**Scheme 46:**
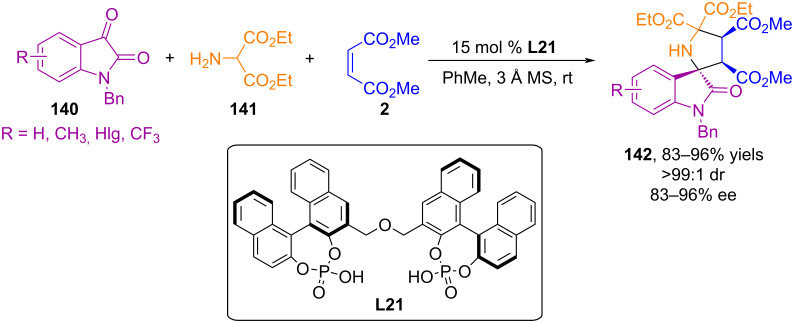
Enantioselective (3 + 2) cycloaddition of azomethine ylides generated in situ from isatins and aminomalonic ester to maleate.

The first example of a 1,3-dipolar cycloaddition reaction involving azomethine ylides generated in situ from isatin and aminomalonic ester and unsaturated carboxylic acids catalyzed by chiral phosphoric acid (**L21**) was reported in [95] (Scheme 46). The method provides a unique platform for the preparation of spiro systems with the simultaneous creation of multiple stereogenic centers. Theoretical calculations showed that in the transition state, the dipole and dipolarophile are simultaneously activated by bisphosphoric acid, forming a chiral catalytic cell in which the cycloaddition occurs.

**Scheme 47:**
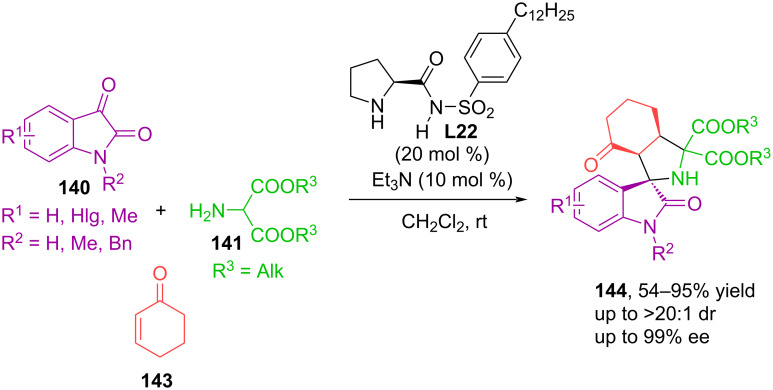
(3 + 2) Cycloaddition of cyclohexenone **143**, isatins **140** and aminomalonic diesters **141**, catalyzed by prolinosulfonamide **L22**.

In 2014, an article [96] was published that examined the enantioselective 1,3-dipolar cycloaddition of cyclohexenone **143** to azomethine ylides obtained in situ from isatins **140** and aminomalonic diesters **141**, catalyzed by readily available proline sulfonamide **L22** (Scheme 47). Spirooxindoles **144** were obtained in high yields (up to 95%) and excellent stereoselectivity (up to 99% ee). The used catalyst **L22** can effectively activate cyclohexenone through the formation of an iminium cation and promote the formation of hydrogen bonds between the catalyst and the dipole [96].

**Scheme 48:**
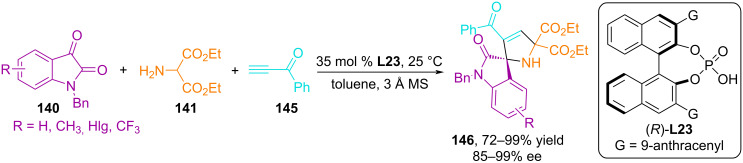
Enantioselective (3 + 2) cycloaddition of azomethine ylides generated in situ from isatins and aminomalonic ester to 1-phenylprop-2-yn-1-one.

The Shi group has successfully implemented the catalytic asymmetric 1,3-dipolar cycloaddition of alkynes to azomethine ylides derived from isatin and diethyl 2-aminomalonate in the presence of BINOL-based chiral phosphoric acid **L23**, affording synthetically and pharmaceutically important spiro[indoline-3,2'-pyrroles] in high yields and excellent enantioselectivity (Scheme 48) [97]. A similar spirocyclic system was constructed via enantioselective (3 + 2) cycloaddition of isatin-derived azomethine ylides to 2,3-allenoates [98]. The authors consider allene in the cycloaddition reaction as a synthetic equivalent of alkyne for constructing the structure of spiro[indoline-3,2'-pyrrole].

**Scheme 49:**
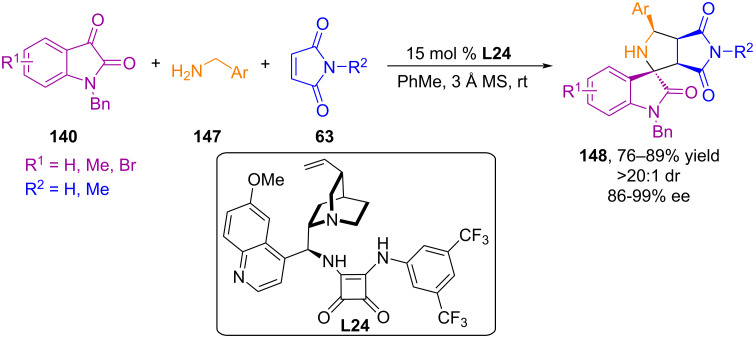
Enantioselective (3 + 2) cycloaddition of azomethine ylides generated in situ from isatins and benzylamines to maleimides.

In a study by Zhao and co-workers, asymmetric 1,3-dipolar cycloaddition of azomethine ylides derived from isatin and benzylamines to maleimides was catalyzed by Cinchona alkaloid-based squaramide **L24** (Scheme 49) [99]. The cycloaddition proceeded facilely, providing pharmaceutically important pyrrolidine-fused spirooxindoles in good yields (up to 89%) and excellent diastereo- and enantioselectivity (up to >20:1 dr, >99% ee). The study established an important fact: acidic additives, while not significantly affecting the chemical yield and diastereoselectivity of the reaction, have a significant impact on enantioselectivity. The same bifunctional squaramide **L24** showed better efficiency as a catalyst for the (3 + 2) cycloaddition reaction of azomethine ylides from isatin and benzylamines to nitroalkenes [100].

**Scheme 50:**
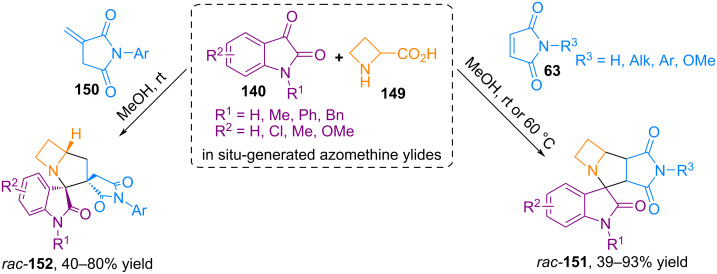
(3 + 2) Cycloaddition involving isatins, azetidine-2-carboxylic acid, maleimides or itaconimides.

A wide variety of heterocyclic systems have been created based on azomethine ylides obtained from isatin via the decarboxylative route. In 2025, an article [101] was published in which we studied in detail the diastereo- and regioselective three-component (3 + 2) cycloaddition reactions of azomethine ylides, obtained in situ from isatins **140** and azetidine-2-carboxylic acid **149**, with various maleimides **63** and itaconimides **150** (Scheme 50).

DFT calculations showed that the 1,3-dipolar cycloaddition of azomethine ylide, obtained from isatin and azetidine-2-carboxylic acid, to maleimides and itaconimides occurs via a concerted mechanism. The formation of regio- and *endo*-stereoselective spiro- and dispiro-derivatives of azabicyclo[3.2.0]heptane **151** and **152** is due to orbital control along with second orbital interactions [101].

**Scheme 51:**
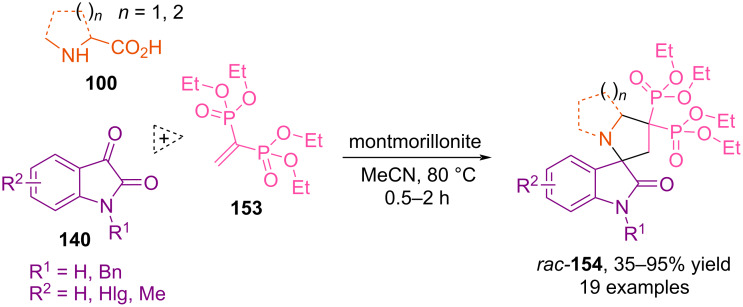
(3 + 2) Cycloaddition involving isatins, amino acids and tetraethylvinylidenebis(phosphonate).

In 2015, Wu and co-workers described a multicomponent synthesis of various spiro[indole-pyrrolizine], spiro[indole-indolizine], and spiro[indole-pyrrolidine] *gem*-bisphosphonates **154** via reactions between substituted isatins **140**, tetraethylvinylidenebis(phosphonate) **153**, and amino acids **100** in the presence of montmorillonite (Scheme 51) [102].

Although this reaction proceeds moderately without a catalyst, the introduction of montmorillonite, a mild Lewis acid, accelerates the process and yields the best yield of the target product. The authors note that the presence of two electron-withdrawing phosphonate groups in the dipolarophile facilitates 1,3-dipolar cycloaddition reactions with azomethine ylides generated from isatins and amino acids, in which the nucleophilic center is activated due to the possibility of delocalization of the negative charge involving the indole ring. In these reactions, the authors used ʟ-proline, piperidine-2-carboxylic acid, and sarcosine. In all cases, the synthesis proceeds with high regioselectivity, forming cycloadducts in yields of up to 95%.

**Scheme 52:**
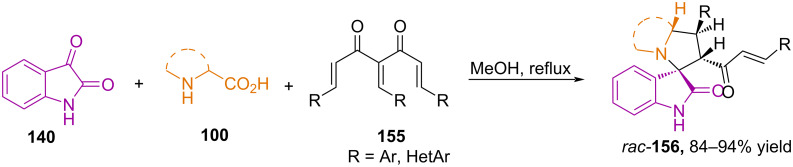
Synthesis of spirooxindoles **156** from triarylideneacetylacetones **155**.

In work [103], the 1,3-dipolar cycloaddition reactions of stabilized azomethine ylides, obtained by decarboxylative condensation of isatin **140** with sarcosine/ʟ-proline/octahydro-1*H*-indole-2-carboxylic acid, with various derivatives of triarylidene acetylacetone **155** were studied (Scheme 52).

It was established that the cycloaddition proceeds through an *endo* transition state and forms a syn-*endo* cycloadduct, while the possibility of forming another isomer through an *exo* transition state is unlikely. The reaction proceeds chemoselectively at the most electron-deficient central multiple bond of triarylidene acetylacetone. The cleavage of the cinnamoyl group that occurs during the formation of spiroheterocycles was additionally confirmed using mass spectrometry [103].

**Scheme 53:**
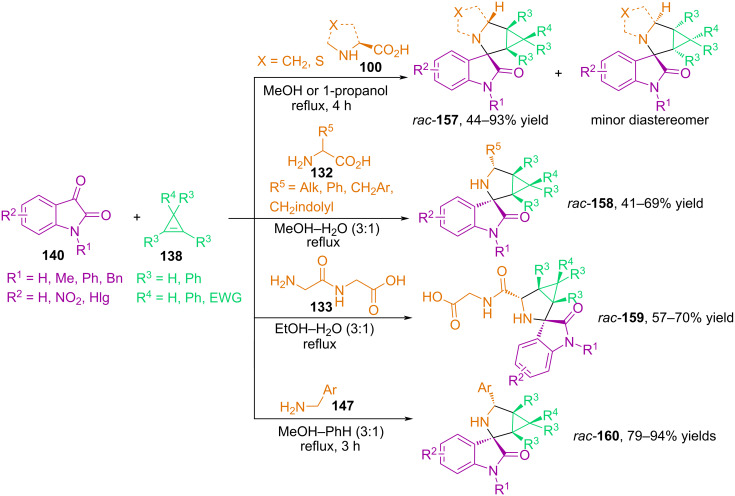
Synthesis of spirooxindole derivatives **157**–**160**.

In [104–105], our group investigated three-component one-pot reactions of substituted and unsubstituted isatins, various α-amino acids, as well as the peptide Gly–Gly and benzylamines with cyclopropenes, which resulted in the preparation of 3-spiro[cyclopropa[*a*]pyrrolizine]- and 3-spiro[3-azabicyclo[3.1.0]hexane]oxindoles **157**–**160**, predominantly in the form of one diastereomer, with yields of up to 94% (Scheme 53). In 2019, the Kanizsai group described the regio- and diastereoselective 1,3-dipolar cycloaddition of 2*H*-azirines to azomethine ylides generated in situ from isatins and α-amino acids, resulting in a previously unknown aziridine-fused spiro[imidazolidine-4,3′-oxyindole] scaffold [106].

**Scheme 54:**
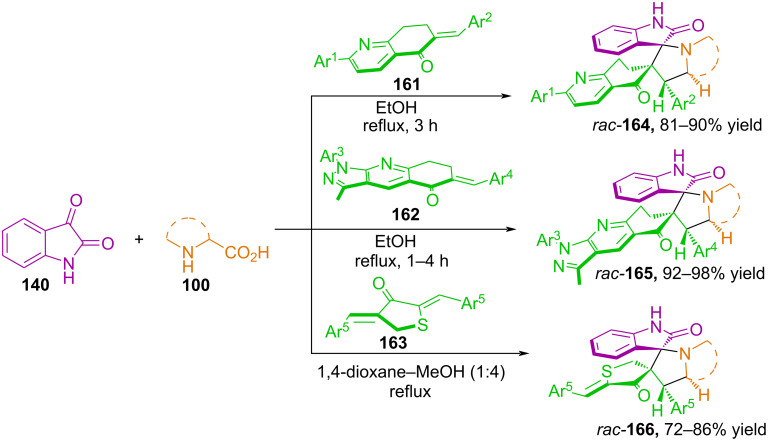
Synthesis of hybrid spiro-heterocycles **164**–**166**.

In [90,107–108], Kumar and co-workers developed a regio- and stereoselective method for the synthesis of dispirooxindole-pyrrolidine derivatives **164–166** based on (3 + 2) cycloaddition reactions involving azomethine ylides based on isatin and *exo*-cyclic alkenes (Scheme 54).

**Scheme 55:**
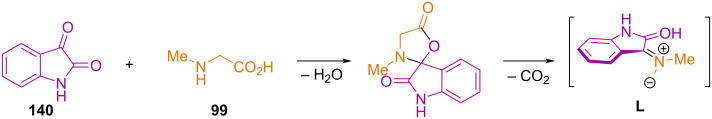
Formation of azomethine ylide from isatin and sarcosine.

In these studies, the authors used dipolarophiles such as dihydroquinolin-5(6*H*)-ones **161**, 6-arylidene-*N*-aryl-pyrazolo[3,4-b]quinolin-5-ones **163**, and 2,4-bis(arylidene)dihydrothiophen-3(2*H*)-ones **164**, which, when reacted with azomethine ylides based on isatin and secondary α-amino acids, such as sarcosine, thiaproline, and pipecolic acid, form spiro-fused products **164**–**166** with four new adjacent stereocenters in yields of up to 98%. When discussing the proposed reaction mechanism, the authors concluded that the initial interaction of isatin and sarcosine proceeds via decarboxylation to form azomethine ylide **L** (Scheme 55).

Further cycloaddition of the *exo-*cyclic alkene proceeds predominantly with the formation of a dispirocycloadduct, in which the carbonyls of the dipole and dipolarophile are in the *trans* position. The authors note that the *cis* position of the carbonyls is undesirable due to electrostatic repulsion. The regioselectivity of the reaction is explained by the polarization of the C=C bond of the *exo*-cyclic alkene, in which the more electron-deficient β-carbon atom reacts with the electron-rich carbon of the 1,3-dipole, forming the corresponding cycloadduct [107].

**Scheme 56:**
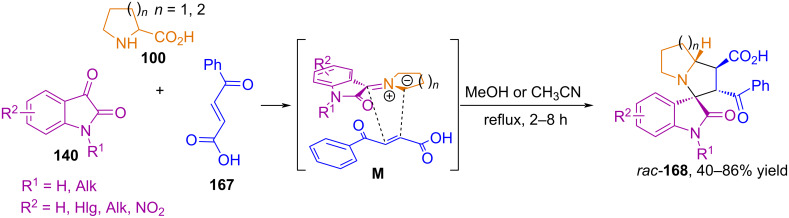
(3 + 2) Cycloaddition involving isatins, amino acids and *trans*-3-benzoylacrylic acid.

In 2025, Quiroga and Abonia described a regio- and stereoselective three-component synthesis of pyrrolizine- and indolizine-spirooxindole derivatives **168** via (3 + 2) cycloaddition of isatins, α-amino acids, and *trans*-3-benzoylacrylic acid **167** (Scheme 56) [109]. The authors found that cycloaddition proceeds through the sterically least hindered transition state **M**, and of the four possible products, predominantly one regio- and stereoisomer **168** is formed.

**Scheme 57:**
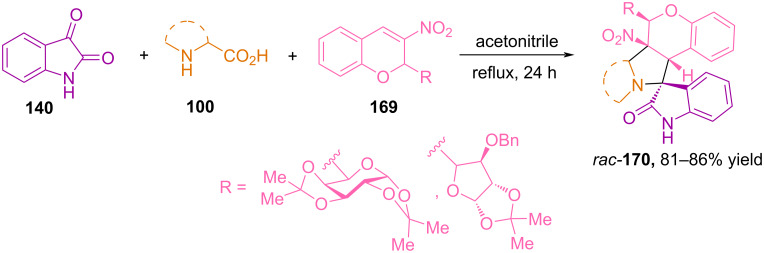
Regioselective synthesis of spirooxindoles **170**.

In 2015, Rao and Raghunathan reported the synthesis of glyco-3-nitrochromane hybrid spiroheterocycles **170** via (3 + 2) cycloaddition of azomethine ylides, prepared in situ from isatin and various amino acids, to 3-nitrochromenes **169** modified with a carbohydrate moiety [110] (Scheme 57). It was established that glyco-3-nitrochromenes react with azomethine ylides based on isatin and various amino acids (sarcosine, proline, pipecolic acid), forming glyco-3-nitrochromane-hybrid pyrrolidinylspirooxindoles with yields from 82 to 86% and high regioselectivity.

**Scheme 58:**
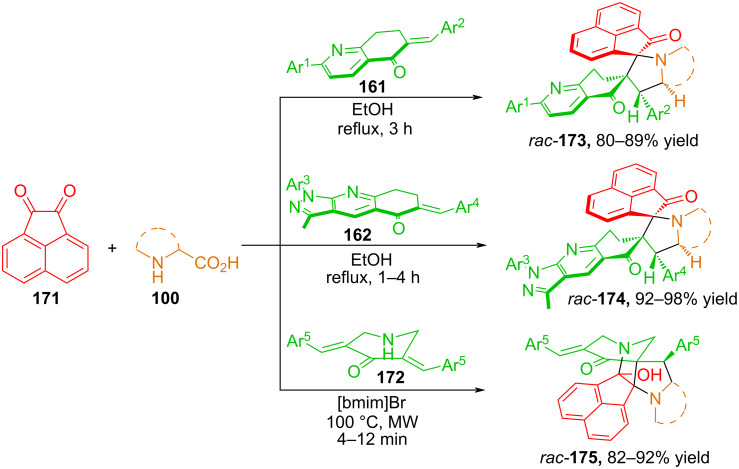
Synthesis of hybrid spiro-heterocycles **86**.

**Azomethine ylides from acenaphthenequinone**. In [107–108,111], Kumar and co-workers studied the 1,3-dipolar cycloaddition reactions of azomethine ylides based on acenaphthylene-1,2-dione **171** with *exo*-cyclic alkenes **161**, **162**, and **172** (Scheme 58). It was established that the interaction of 6-arylidene-2-aryl-7,8-dihydroquinolin-5(6*H*)-ones **161** and 6-arylidene-*N*-aryl-pyrazolo[3,4-b]quinolin-5-ones **162** with azomethine ylides formed in situ as a result of decarboxylative condensation of acenaphthenequinone and secondary α-amino acids such as sarcosine, thiaproline, and pipecolic acid results in the formation of bis-spirocycloadducts **173** and **174** in high yields, as well as with high stereo- and regioselectivity [107–108].

The cycloaddition of *exo*-cyclic alkene **172** occurred in the ionic liquid 1-butyl-3-methylimidazolinium bromide [bmim]Br under microwave activation conditions. Proline and phenylglycine were used as amino acids. The addition products **175** were obtained in high yields and with high stereo- and regioselectivity [111].

**Scheme 59:**
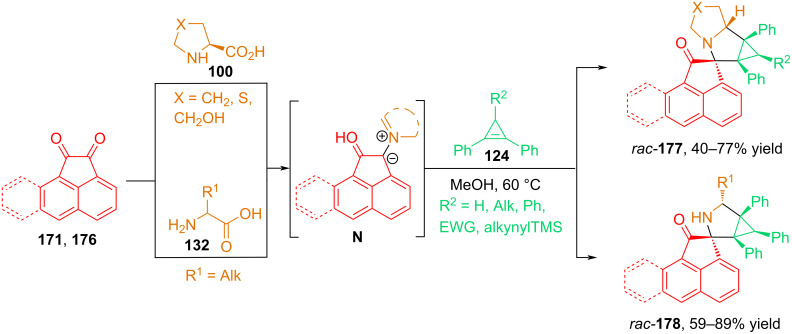
(3 + 2) Cycloaddition involving acenaphthenequinones, amino acids and cyclopropenes.

In 2024, our group described the (3 + 2) cycloaddition of azomethine ylides based on acenaphthylene-1,2-dione with cyclopropene dipolarophiles [112]. Based on the reaction, a simple and efficient method was developed for the preparation of cyclopropa[*a*]pyrrolizidines **177** and 3-azabicyclo[3.1.0]hexanes **178** spiro-fused with an acenaphthylene-1,2-dione or aceanthrylene-1,2-dione moiety via the reaction of diketones with α-amino acids and 1,2-diphenylcyclopropenes **124** (Scheme 59).

The authors examined the influence of the electronic nature of the substituents at position C^3^ of 1,2-diphenylcyclopropenes on the yields of the reaction products. It was found that the highest yields (68–77%) were obtained for cycloadducts obtained using cyclopropenes without substituents at position 3 or with substituents such as phenyl, vinyl, and TMS-ethynyl. However, when using 1,2-diphenylcyclopropenes with electron-withdrawing substituents such as carboxymethyl and nitrile, the yields of the products decreased to 44 and 40%, respectively. To generate azomethine ylides from acenaphthenequinone, the authors used ʟ-proline and its derivatives, such as ʟ-hydroxyproline and thiazolidine-4-carboxylic acid, as well as acyclic α-amino acids, such as norvaline, norleucine, leucine, and methionine.

To determine the reasons for the high diastereoselectivity in these reactions, comprehensive computational studies using density functional theory (DFT) were conducted. Based on the data obtained, it was concluded that the cycloaddition, with the formation of predominantly one *endo*-diastereomer, is due to the energetically more favorable approach of the S-ylide to 1,2-diphenylcyclopropene via the *endo*-transition state.

**Scheme 60:**
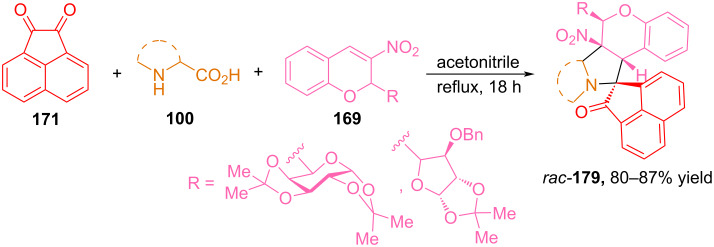
Synthesis of hybrid glyco-3-nitrochromane cycloadducts **179**.

In addition to using isatin to generate azomethine ylides in reactions with glyco-3-nitrochromenes **169**, Rao and Raghunathan studied the possibility of using acenaphthenequinone **171** for this process (Scheme 60) [110]. It was found that the interaction of **171**, **169** and various amino acids formed glyco-3-nitrochromane cycloadducts **179** in yields from 80 to 87% and with high regioselectivity.

**Scheme 61:**
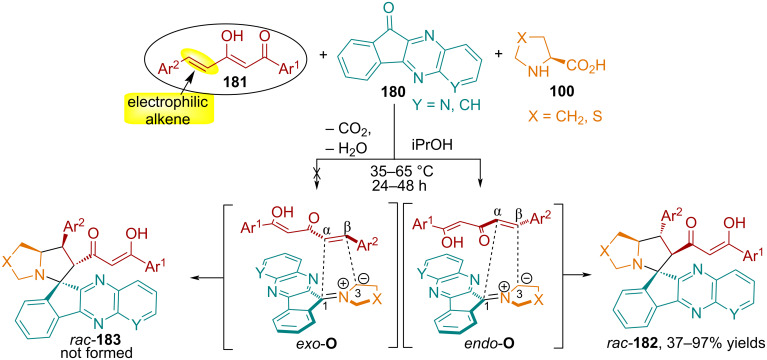
Synthesis of spiro[indenoquinoxaline-(thia)pyrrolizidines] **90a**.

**Azomethine ylides from 11*H*-indeno[1,2-*b*]quinoxalin-11-one**. In [113], Sosnovskikh and co-workers searched for suitable dipolarophiles that would react smoothly with azomethine ylides obtained from indenoquinoxalinone **180**. For example, the relatively easily accessible (*E*)-1,5-diarylpent-4-ene-1,3-diones **181**, also called hemicurcuminoids or 5C-curcuminoids, hold special significance as electrophilic alkenes due to their incorporation of a 1,3-diketone moiety. It was established that 1,3-dipolar cycloaddition based on 11*H*-indeno[1,2-*b*]quinoxalin-11-ones and α-amino acids stabilized azomethine ylides to dipolarophiles **181** proceeds regio- and stereoselectively with the formation of spiro[indenoquinoxaline-(thia)pyrrolizidines] **182** in yields from 37 to 97% (Scheme 61).

The authors propose that the reactions of 1,3-diones with stabilized azomethine ylides derived from indenoquinoxalinones and cyclic amino acids proceed via attachment of the more electrophilic β-C atom of the dipolarophile to the less substituted C^3^ atom of the 1,3-dipole, likely governed by orbital control of the cycloaddition process. In the *exo*-O transition state, the 1,3-dicarbonyl and quinoxaline units, together with the Ar^2^ substituent and the (thia)proline ring, are positioned directly above one another, resulting in reduced stability due to steric repulsion. Thus, the reactions proceed with the formation of *endo*-cycloadducts, the structure and relative configuration of which were unambiguously established using X-ray structural analysis [113].

**Scheme 62:**
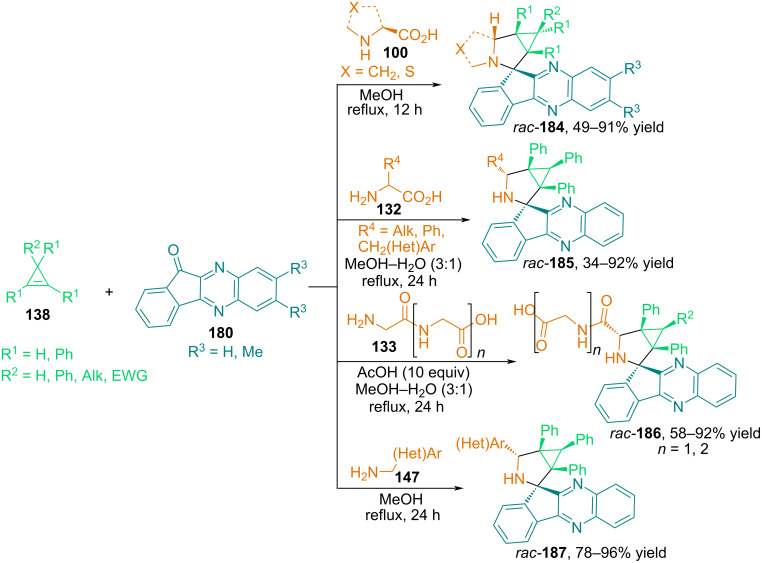
Three-component reactions of cyclopropenes, 11*H*-indeno[1,2-*b*]quinoxalin-11-onesand α-amino acids, simple peptides or benzylamines.

In [114], we described in detail the 1,3-dipolar cycloaddition of various cyclopropenes and azomethine ylides obtained in situ from 11*H*-indeno[1,2-*b*]quinoxalin-11-ones **180** and primary or secondary α-amino acids, simple peptides and benzylamines (Scheme 62). A unique feature of this work is that reactions using 11*H*-indeno[1,2-*b*]quinoxalin-11-ones can involve a fairly wide range of primary α-amino acids, including such rarely used substrates as tyrosine, 3,5-diiodotyrosine, tryptophan, histidine, serine, cysteine, homoserine, arginine, asparagine, and glutamine. In addition to the above-mentioned amino acids, simple peptides (Gly–Gly and Gly–Gly–Gly) and benzylamines, such as 4-methylbenzylamine, 4-fluorobenzylamine, 3-picolylamine, and 2-furylmethylamine, can also participate in cycloaddition. 1,2-Diphenylcyclopropene and 1,2-diphenylcyclopropenes with electron-donating (Ph, Et, vinyl) and electron-withdrawing (CO_2_Me, CONHiPr, CN) substituents at position C^3^ showed good results in the reactions. The resulting 3-azabicyclo[3.1.0]hexanes and cyclopropa[*a*]pyrrolizines, spiro-fused with an indeno[1,2-*b*]quinoxaline fragment, exhibit good yields and high diastereoselectivity.

**Scheme 63:**
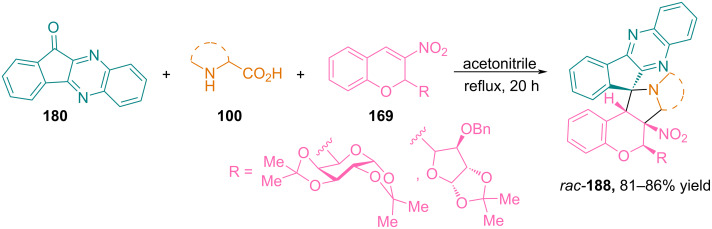
Synthesis of hybrid glyco-3-nitrochromane cycloadducts **92**.

In [110], Rao and Raghunathan considered the possibility of using indenoquinoxalinone **180** as a precursor of azomethine ylide in reactions with 3-nitrochromenes **169** modified with a carbohydrate substituent (Scheme 63).

**Scheme 64:**
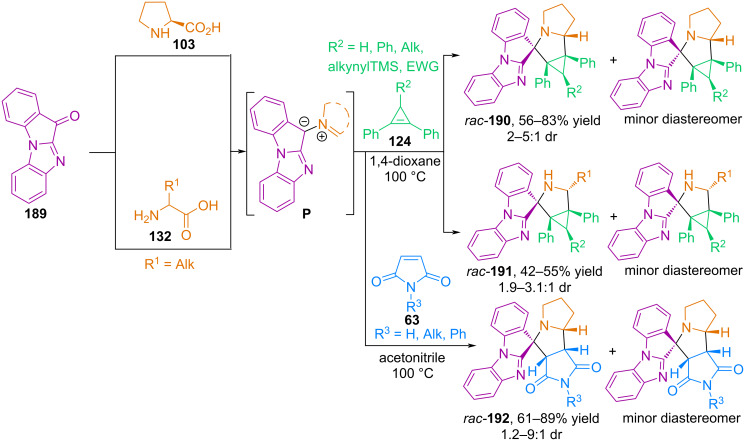
(3 + 2) Cycloaddition of 11*H*-benzo[4,5]imidazo[1,2-*a*]indol-11-one (**189**) with cyclopropenes and maleimides.

**Azomethine ylides from other cyclic ketones**. Our research group is searching for new carbonyl compounds as precursors for the generation of azomethine ylides and studying their reactions with cyclopropene dipolarophiles. In 2022, the authors demonstrated the possibility of generating azomethine ylides from the tetracyclic ketone 11*H*-benzo[4,5]imidazo[1,2-*a*]indol-11-one (**189**) and α-amino acids, and also examined their (3 + 2) cycloaddition to diphenylcyclopropenes **124** and *N*-substituted maleimides **63** (Scheme 64) [115].

A disadvantage of reactions using ketone **189** is the formation of products **190**–**192** as mixtures of two diastereomers that differ in the configuration of the spiro atom. In this synthesis, the authors used cyclopropenes with both electron-donating and electron-withdrawing substituents at position C^3^; in addition to ʟ-proline, the authors introduced 2-aminobutanoic acid, ᴅʟ-norvaline, ʟ-methionine, ᴅʟ-norleucine, and ʟ-leucine as α-amino acids [115].

**Scheme 65:**
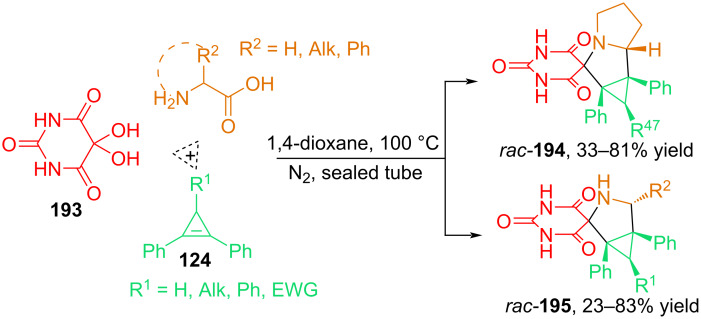
Diastereoselective synthesis of spiro derivatives of barbituric acid from alloxan **193**, α-amino acids and cyclopropenes.

In [2,116], the authors described the 1,3-dipolar cycloaddition of azomethine ylides generated from alloxan **193** and primary or secondary α-amino acids to cyclopropene dipolarophiles **124** (Scheme 65). It was established that the cycloaddition of alloxan azomethine ylides to cyclopropenes proceeds with the formation of spiro adducts **194** and **195** with excellent diastereoselectivity. In addition to ʟ-proline, such α-amino acids as glycine, methionine, norleucine, phenylglycine, and norvaline turned out to be suitable reactants in the three-component synthesis of spirobarbiturate-3-azabicyclo[3.1.0]hexanes **96b** with yields of up to 83%.

**Scheme 66:**
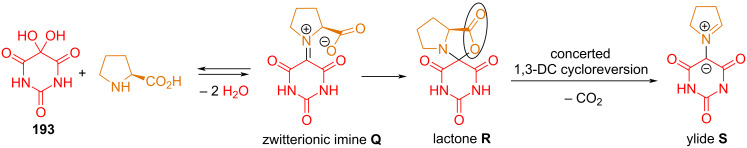
Probable mechanism of formation of azomethine ylide from alloxan and ʟ-proline.

A computational study using density functional theory (DFT) showed that the condensation reaction of alloxan and ʟ-proline leads to the formation of a zwitterionic imine **Q**, which undergoes intramolecular cyclization to form lactone **R** (Scheme 66). Being a thermally unstable compound, the lactone undergoes a decarboxylation reaction via ring opening to form a 1,3-dipole **S**. Thus, the experimentally obtained *endo*-cycloadducts are the most thermodynamically favorable, and the presence of a barbituric acid fragment in the molecule makes the compound biologically significant [2].

**Scheme 67:**
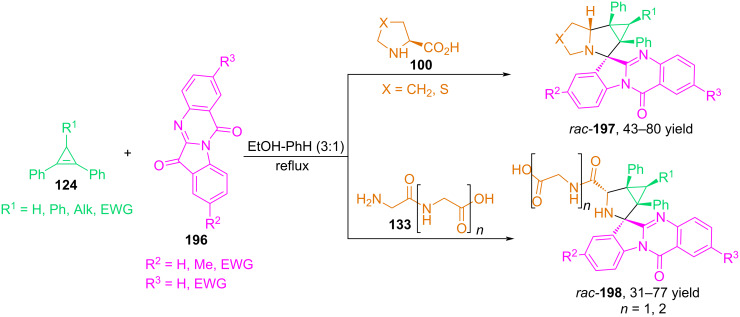
Three-component reactions involving tryptanthrin **196**, α-amino acids and cyclopropenes.

In 2019, our research group published an article on the synthesis of complex alkaloid-like compounds with spiro-fused fragments of indolo[2,1-*b*]quinazoline and cyclopropa[*a*]pyrrolizine or 3-azabicyclo[3.1.0]hexane [117]. The authors found that three-component 1,3-dipolar cycloaddition reactions of azomethine ylides obtained in situ from substituted or unsubstituted tryptanthrins **196** and α-amino acids (ʟ-proline, ʟ-4-thiazolidinecarboxylic acid) or simple peptides (dipeptide Gly–Gly, tripeptide Gly–Gly–Gly) with cyclopropenes containing phenyl substituents at the multiple bond allow the desired products **197** and **198** to be obtained in good yields (up to 80%) and excellent diastereoselectivity (Scheme 67).

The authors decided to examine the effect of substituted tryptanthrins on the reaction. It was shown that the introduction of electron-donating substituents, such as methyl and methoxy groups, at the C^8^ position of tryptanthrin did not result in a significant difference in stereoselectivity compared to unsubstituted tryptanthrin, and spirocycloadducts were obtained in good yields and high diastereoselectivity. However, when tryptanthrins with electron-withdrawing groups (-Cl and -NO_2_) at the C^8^ and C^2^ positions were used in the reactions, the cycloaddition proceeded with a decrease in the yield of products, and a three-component reaction involving 2-nitrotryptanthrin did not lead to the formation of the corresponding cycloadduct at all.

In general, the authors note that azomethine ylides from tryptanthrin and amino acids were less reactive in reactions with cyclopropenes than azomethine ylides based on isatins and 11*H*-indeno[1,2-*b*]quinoxalin-11-ones. For example, three-component reactions involving tryptanthrin, primary amino acids, and cyclopropenes were unsuccessful. The reaction of the azomethine ylide generated from tryptanthrin and ʟ-proline with maleimides is described in [118].

## Conclusion

As can be seen from this review, enantioselective 1,3-dipolar cycloaddition reactions of azomethine ylides based on iminoesters with alkenes of various structures, catalyzed by chiral metal complexes, have now been studied in considerable detail. These reactions provide chemists with reliable methods for the enantioselective synthesis of polysubstituted pyrrolidines. In decarboxylating 1,3-dipolar cycloaddition reactions, special attention is paid to the search for new carbonyl substrates for the generation of azomethine ylides. This is extremely important, as it allows for the production of new cyclic systems of great practical interest. Advances in this field open up new possibilities for the synthesis of complex poly- and spirocyclic compounds. A distinctive feature of such reactions is their high regio- and diastereoselectivity. However, despite significant achievements, unresolved problems remain. For example, azomethine ylides, which are formed by decarboxylating condensation between ketones (or aldehydes) and natural amino acids, are widely used in the synthesis of various biologically active compounds, but their use in asymmetric catalysis has been insufficiently studied. Intensification of work in this direction, in particular the search for new chiral metal complexes capable of coordinating with the nitrogen atom of such azomethine ylides, would contribute to a significant increase in the synthetic significance of this methodology.

In conclusion, it should be noted that through the reactions of 1,3-dipolar cycloaddition of azomethine ylides to various olefins, it becomes possible to create a wide range of unique monocyclic, polycondensed and spiro-fused heterocyclic systems that may be of interest for medicinal chemistry, pharmacology and materials science.

## Data Availability

Data sharing is not applicable as no new data was generated or analyzed in this study.
